# Isolation, structure and reactivity of a scandium boryl oxycarbene complex†
†Electronic supplementary information (ESI) available: Synthesis, characterization of compounds **2** **–** **7**. CCDC 981561–981563, 1042268 and 1042269. For ESI and crystallographic data in CIF or other electronic format see DOI: 10.1039/c5sc03138a


**DOI:** 10.1039/c5sc03138a

**Published:** 2015-09-16

**Authors:** Baoli Wang, Xiaohui Kang, Masayoshi Nishiura, Yi Luo, Zhaomin Hou

**Affiliations:** a Organometallic Chemistry Laboratory and RIKEN Center for Sustainable Resource Science , RIKEN , 2-1 Hirosawa , Wako , Saitama 351-0198 , Japan . Email: houz@riken.jp; b State Key Laboratory of Fine Chemicals , School of Pharmaceutical Science and Technology , Dalian University of Technology , Dalian 116024 , China . Email: luoyi@dlut.edu.cn

## Abstract

The reaction of a half-sandwich scandium boryl complex **1** with CO (1 atm) afforded a novel boryl oxycarbene complex **2**. The structure of **2** was characterized by ^1^H, ^13^C and ^11^B NMR, X-ray diffraction, and DFT analysis. Further reaction of **2** with CO (1 atm) yielded a phenylamido- and boryl-substituted enediolate complex **3** through C–C bond formation between CO and the carbene unit in **2** and cleavage and rearrangement of the Si–N bond in the silylene-linked Cp–amido ligand. Upon heating, insertion of the carbene atom into a methine C–H bond in the boryl ligand of **2** took place to give an alkoxide complex **4**. The reactions of **2** with pyridine and 2-methylpyridine led to insertion of the carbene atom into an *ortho*-C–H bond of pyridine and into a methyl C–H bond of 2-methylpyridine, respectively. The reaction of **2** with ethylene yielded a borylcyclopropyloxy complex **7** through cycloaddition of the carbene atom to ethylene.

## Abstract 2

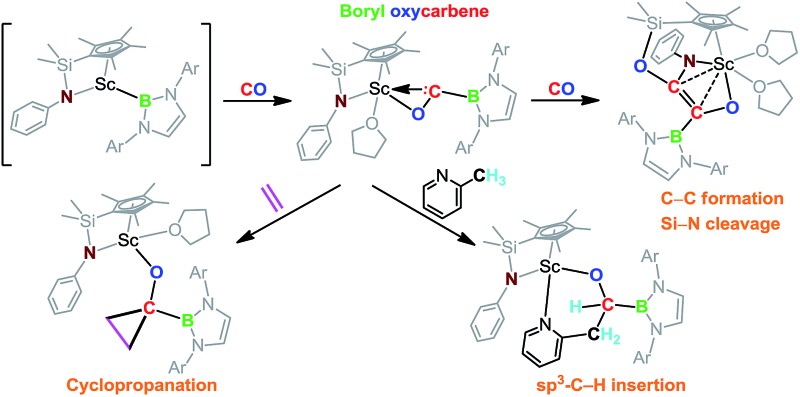
A well-defined scandium boryl oxycarbene complex undergoes coupling with CO, C–H activation of lutidine, and cyclopropanation with ethylene.

## Introduction

Carbon monoxide (CO) is an important C_1_ building block in chemical industry, as it can be used for the production of synthetic lubrication oils and fuels *via* Fischer–Tropsch reactions.^1^ So far, extensive studies on the reaction of CO with transition metal alkyls and hydrides have been reported in relevance to the Fischer–Tropsch process.^1–3^ The reaction of early transition-metal (including lanthanide and actinide) alkyls (or hydrides) with CO usually gives η^2^-acyl (or formyl) species that shows carbene-like characteristics in reactivity such as intramolecular 1,2-hydrogen migration, dimerization, and ketene formation.^3–5^ The analogous reactions of silyl, amido and phosphido complexes of some early transition metals with CO were also reported.^6^ In spite of extensive studies in this area, structurally characterized carbene-like species (or oxycarbene complexes) remains scarce. In 1980, Marks and co-workers reported that the reaction of a sterically demanding bis(pentamethylcyclopentadienyl) thorium neopentyl complex [(C_5_Me_4_)_2_Th{CH_2_C(CH_3_)_3_}Cl] with 1 equivalent of CO could afford a structurally characterizable oxycarbene complex [(C_5_Me_5_)_2_Th{η^2^-OCCH_2_C(CH_3_)_3_}Cl].^4*a*^ This is perhaps the only precedent of a well-defined oxycarbene complex.

Metal boryl complexes have received much attention in the last few decades because of their important roles in various chemical transformations.^7–9^ In this context, the reactions of metal boryl compounds with metal carbonyl complexes were recently reported, such as the nucleophilic addition of [(THF)_2_Li{B(NDippCH)_2_}] (Dipp = 2,6-diisopropylphenyl) to [Fe(CO)_5_] and [Cr(CO)_6_] as well as the intramolecular migratory addition of a boryl ligand to a carbonyl group in [(CO)_4_Co{B(NDippCH)_2_}].^10a,b^ The reaction of metal carbonyl complexes such as K[(η^5^-C_5_H_5_)M(CO)_3_] (M = Mo, W) with B_2_(NMe_2_)_2_I_2_ to give oxycarbyne complexes was also reported.^10c–e^ In contrast, the reaction of gaseous CO with metal boryl compounds remains much less extensively explored.^9*b*^

In 2011, we reported the reaction of a bis(amidinate)-ligated rare-earth boryl complex [{(Me_3_SiCH_2_)C(N^*i*^Pr)_2_}_2_Sc{B(NDippCH)_2_}] with gaseous CO (1 atm), which afforded a double CO insertion product. This reaction was proposed to proceed through a scandium borylacyl (or carbene) intermediate, but the isolation of such an acyl (or carbene) species was not achieved.^9*b*^ More recently, we found that a half-sandwich structure unit with a silylene-linked Cp–anilido ligand could serve as a useful platform for the isolation and transformation of rare-earth boryl species such as [Me_2_Si(C_5_Me_4_)(NPh)Sc{B(NDippCH)_2_}(μ-Cl)Li(THF)_3_] (**1**).^9*c*^ In this paper, we report the isolation and structural characterization of a boryl oxycarbene complex [Me_2_Si(C_5_Me_4_)(NPh)Sc{η^2^-OCB(NDippCH)_2_}(THF)] (**2**) formed by reaction of the half-sandwich scandium boryl complex **1** with CO. The diverse reactivity of the boryl oxycarbene complex **2**, such as intra- and intermolecular sp^2^ and sp^3^ C–H bond insertion, cyclopropanation with ethylene, and C–C bond formation with another molecule of CO is also described.

## Results and discussion

### Isolation and structure of a scandium boryl oxycarbene complex

**Scheme 1:**
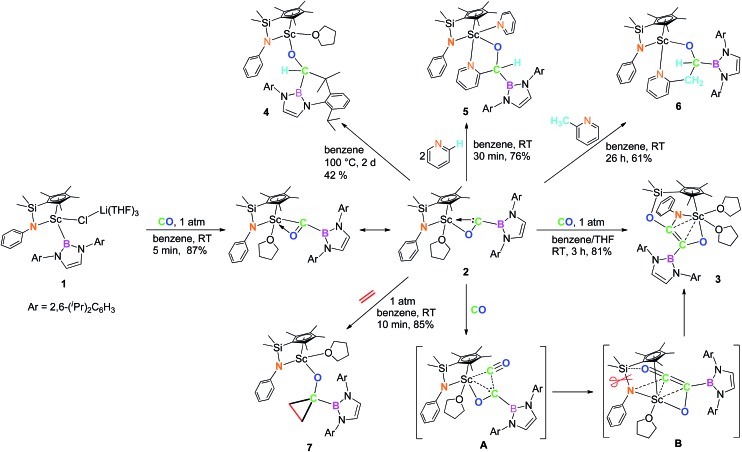
Synthesis and reactivity of the boryl oxycarbene scandium complex **2**.

When the half-sandwich scandium boryl (**1**) was exposed to a CO atmosphere (1 atm) at room temperature in benzene-d_6_, the insertion of CO into the Sc–boryl bond took place rapidly, selectively yielding the corresponding scandium borylacyl (or oxycarbene) complex **2** in 87% yield as dark blue crystals within 5 min (Scheme 1). The (THF)_3_LiCl adduct in **1** is dissociated in this reaction. The reaction of **1** with ^13^C-enriched CO afforded the ^13^C-labeled analogue **2-^13^C** (eqn (1)). The ^13^C NMR spectrum of **2** (or **2-^13^C**) in benzene-d_6_ gave a singlet at *δ* 427.4 assignable to the CO group. This signal is considerably downfield shifted than those of reported transition-metal acyl complexes (*δ* 214.4–322.9),^11^ and even lower than that of the thorium oxycarbene complex [(C_5_Me_5_)_2_Th{η^2^-OCCH_2_C(CH_3_)_3_}Cl] (*δ* 360.2),^4*a*^ clearly demonstrating the presence of a carbene species. The ^11^B{H} NMR of **2** in benzene-d_6_ showed a singlet at *δ* 16.9, which is 6.4 ppm up-field shifted from that of a cobalt Fischer-type boryl oxycarbene complex [(OC)_5_Cr{C(OEt)B(NDippCH)_2_}] (*δ* 23.3).^10*a*^

**Fig. 1:**
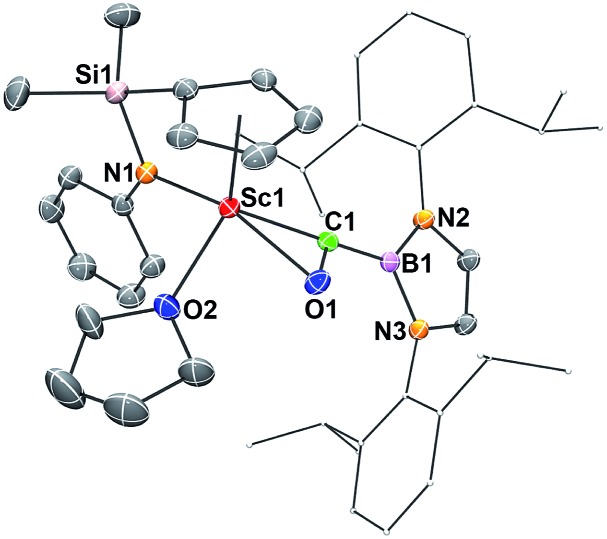
ORTEP drawing of **2** with thermal ellipsoids at the 30% level except for the 2,6-(^*i*^Pr)_2_C_6_H_3_ groups in the boryl unit. Hydrogen atoms and the Me groups on the Cp ring have been omitted for clarity. Selected bond lengths (Å) and angles (°): Sc1–N1 2.130(2), Sc1–O1 2.114(2), Sc1–O2 2.199(2), Sc1–C1 2.194(2), C1–O1 1.266(3), C1–B1 1.577(3); Sc1–C1–O1 69.48(12), Sc1–O1–C1 76.40(12), Sc1–C1–B1 172.11(16).

Single crystals of **2** suitable for X-ray diffraction studies were obtained by recrystallization from a mixed hexane–benzene solution at –30 °C. An X-ray diffraction study revealed that the Sc atom is bonded to the CO unit in a η^2^-fashion (Fig. 1). The Sc–O1 bond distance (2.114(2) Å) is significantly shorter than that of the Sc–C1 bond (2.194(2) Å), similar to what was observed in the thorium oxycarbene complex [(C_5_Me_5_)_2_Th{η^2^-OCCH_2_C(CH_3_)_3_}Cl] (Th–O 2.37(2) Å, Th–C 2.44(2) Å).^4*a*^ The C1–O1 bond length (1.266(3) Å) in **2** is longer than that in [(C_5_Me_5_)_2_Th{η^2^-OCCH_2_C(CH_3_)_3_}Cl] (1.18(3) Å),^4*a*^ suggesting that the η^2^-CO unit in **2** is better considered as a carbene moiety than an acyl group.

In order to gain a better understanding about the nature of bonding of the boryl oxycarbene unit in **2**, DFT studies at the M06 level were carried out.^12^ The calculated structure showed excellent agreement with the crystallographic structure, especially for the bond lengths of the Sc1–C1 (2.17 Å *vs.* 2.194(2) Å) and Sc1–O1 (2.12 Å *vs.* 2.114(2) Å) bonds.

**Fig. 2:**
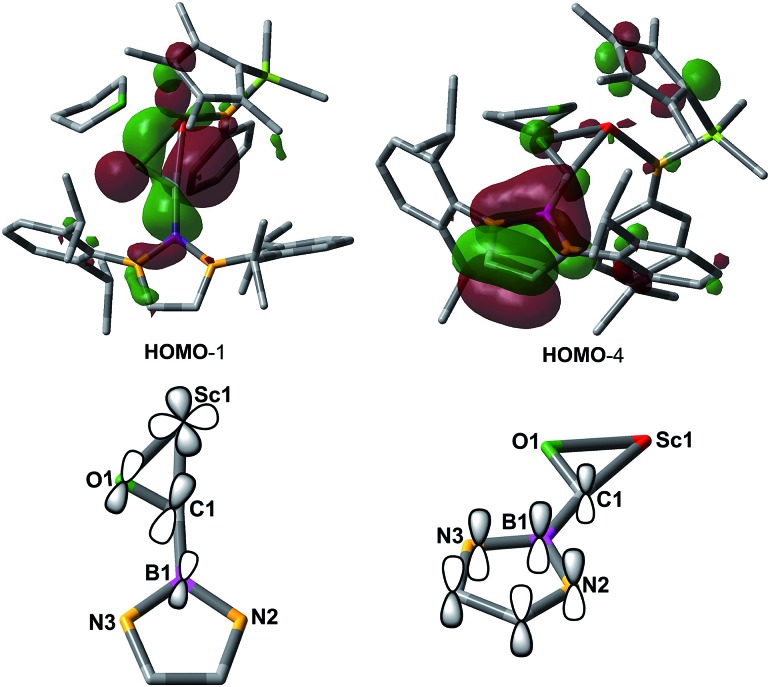
Selected molecular orbitals for **2** (all H atoms are omitted for clarify).

The C1–O1 stretching frequency of **2** is difficult to assign experimentally due to overlapping bands with those of the boryl moiety. The computed C1–O1 stretching frequencies of **2** (1450 cm^–1^) and **2-^13^C** (1417 cm^–1^) are comparable with the experimental IR values of the thorium oxycarbene complex [(C_5_Me_5_)_2_Th{η^2^-OCCH_2_C(CH_3_)_3_}Cl] (1469 cm^–1^) and its ^13^CO analogue (1434 cm^–1^), which are lower than those of transition-metal acyl complexes (1523–1666 cm^–1^).^11^ Further molecular orbital analysis of **2** suggests significant Sc1–O1 and Sc1–C1 bonding interactions with a minor contribution from the B1-2p orbital (see HOMO–1 in Fig. 2). HOMO–4 indicates π–bonding between C1, B1 and two N atoms of the boryl moiety (Fig. 2). The analysis of the donor–acceptor interactions on the basis of second-order perturbation theory^13^ revealed that the donation of σ(B1–C1) to Sc1 (177.7 kcal mol^–1^) is significantly stronger than that of σ(O1–C1) (96.0 kcal mol^–1^), and the donation of lone pair electrons of C1 to a vacant 3d orbital of Sc1 (170.3 kcal mol^–1^) is higher than that of O1 to Sc1 (83.3 kcal mol^–1^). In addition, the donation of the lone pair electrons of N2 (88.8 kcal mol^–1^) and N3 (57.9 kcal mol^–1^) atoms to B1 was also found in the boryl segment. Therefore, The boryl group plays an important role in stabilizing the Sc–(boryl)carbene moiety.
1

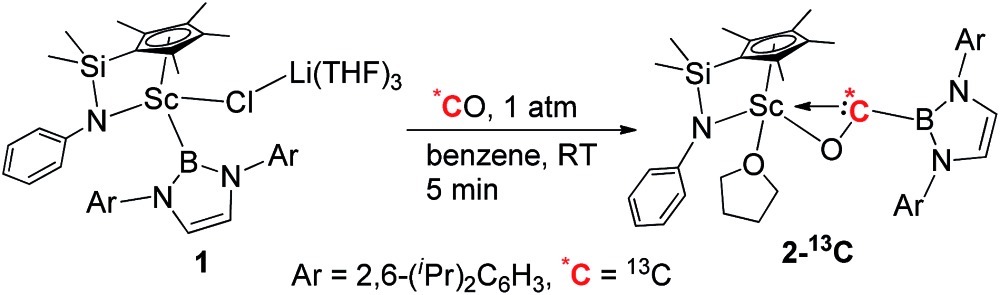




### Reaction of carbene with CO

**Fig. 3:**
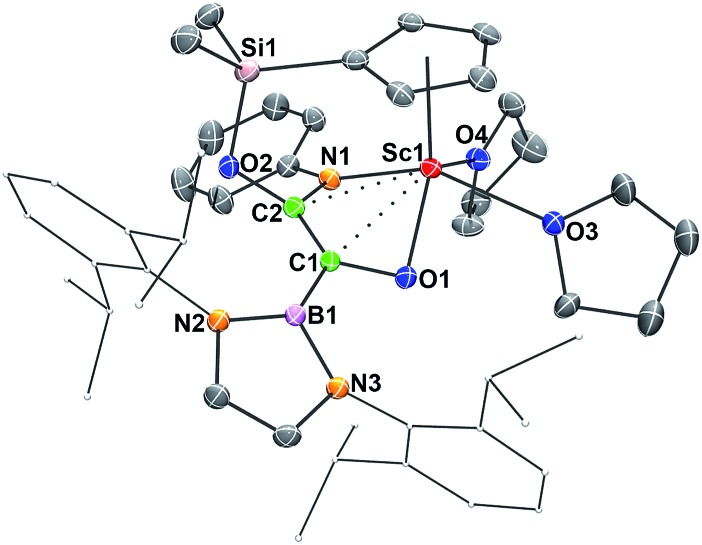
ORTEP drawing of **3** with thermal ellipsoids at the 30% probability except for the 2,6-(^*i*^Pr)_2_C_6_H_3_ groups in the boryl unit. Hydrogen atoms have been omitted for clarity. Selected bond lengths (Å): Sc1–N1 2.086(3), Sc1–O1 2.005(2), Sc1–C1 2.519(3), Sc1–C2 2.477(3), Sc1–O3 2.335(3), Sc1–O4 2.291(2), C1–C2 1.364(5), C1–O1 1.383(4), C2–O2 1.390(4), C2–N1 1.405(4), B1–C1 1.567(5), Si1–O2 1.691(2).

When being exposed to CO (1 atm) in a benzene–THF solution at room temperature for 3 h, **2** was completely consumed, and a phenylamido- and boryl-substituted enediolate complex **3** was obtained in 81% yield as yellow crystals after crystallization from a hexane–benzene solution (Scheme 1). An X-ray crystallographic study established that C–C bond formation between the carbene atom in **2** and CO occurred, accompanied by cleavage of the Si–NPh bond and formation of the O2–Si and C2–NPh bonds (Fig. 3). The resulting C1–C2 bond in **3** could be assigned as a double bond (1.364(5) Å), which shows some interactions with the Sc atom (Sc1–C1 2.519(3) Å, Sc1–C2 2.477(3) Å). The two oxygen atoms (O1 and O2) attached to the C1=C2 double bond are *trans* to each other, so are the boryl and PhN groups.

To further confirm the formation of **3**, the ^13^C-enriched complexes **3-^13^C**, **3-^13^C′** and **3-^13^C_2_** were synthesized analogously, as shown in eqn (2)–(4). The ^13^C NMR spectrum of **3-^13^C_2_** in benzene-d_6_ showed a broad doublet at *δ* 134.6 and a sharp doublet at *δ* 136.7 for the OC=CO unit, whilst the ^13^C NMR spectra of **3-^13^C** and **3-^13^C′** gave a singlet at *δ* 136.7 and *δ* 134.7, respectively. The ^11^B{H} NMR signal of **3** appeared at *δ* 23.8, which was 6.9 ppm downfield shifted compared to that of **2** (*δ* 16.9).
2

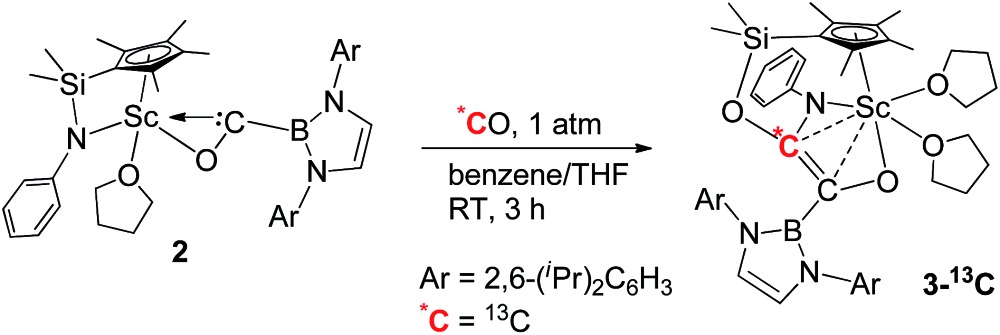



3

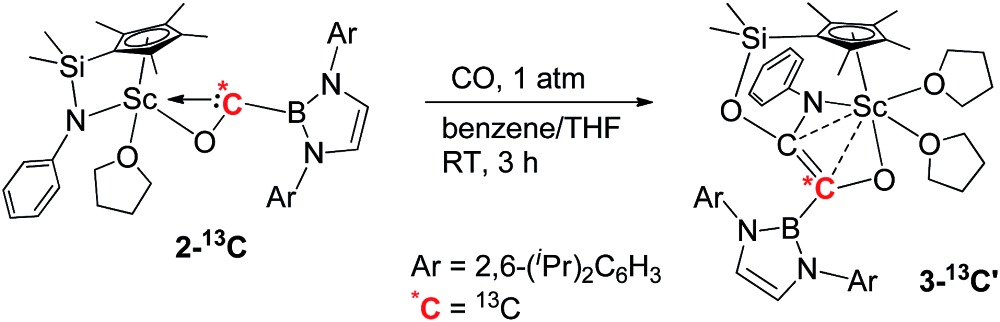



4

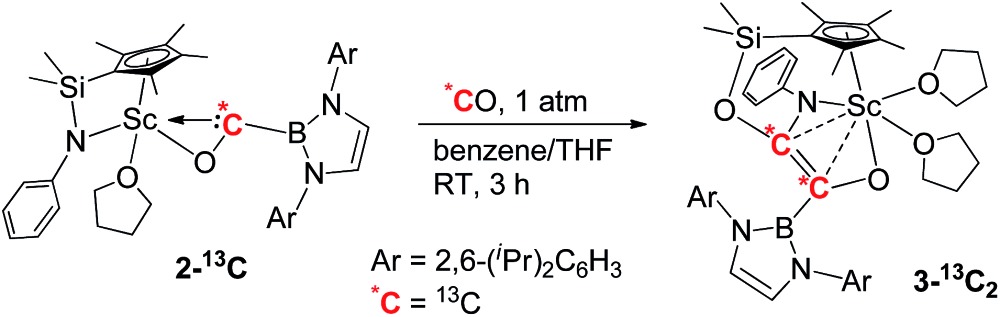




The formation of **3** may be achieved by insertion of CO into the Sc–carbene bond in **2** to give a ketene unit,^14a–c^ followed by cleavage of the Si–N bond in the Cp–anilido ligand and formation of a Si–O bond and an N–C bond between the resulting silyl and PhN groups and the OCCO unit (*cf.* **A** and **B** in Scheme 1). Silylene-linked Cp–amido ligands have been used for the stabilization of various metal complexes, but examples of cleavage of the Si–N bond in these ligands are scarce.^15^ A possible driving force for the present Si–N cleavage could be the formation of stable Si–O and C–N bonds. A similar silyl migration reaction was also observed previously in the reaction of a bis(amidinate)-ligated scandium boryl complex with CO.^9*b*^ The reaction of transition-metal acyl complexes [M–C(=O)R] with CO were previously reported to give α-ketoacyl species such as [M–C(=O)C(=O)R].^14*d*^ The reaction of a metallocene cerium hydride complex Cp′_2_CeH (Cp′ = 1,2,4-(^t^Bu)_3_C_5_H_2_) with CO was reported to yield an enediolate complex [Cp′_2_CeOCH=CHOCeCp′_2_] without observation of an isolable mono-CO insertion product.^5*l*^

### Intra- and intermolecular insertion of carbene into C–H bonds

**Fig. 4:**
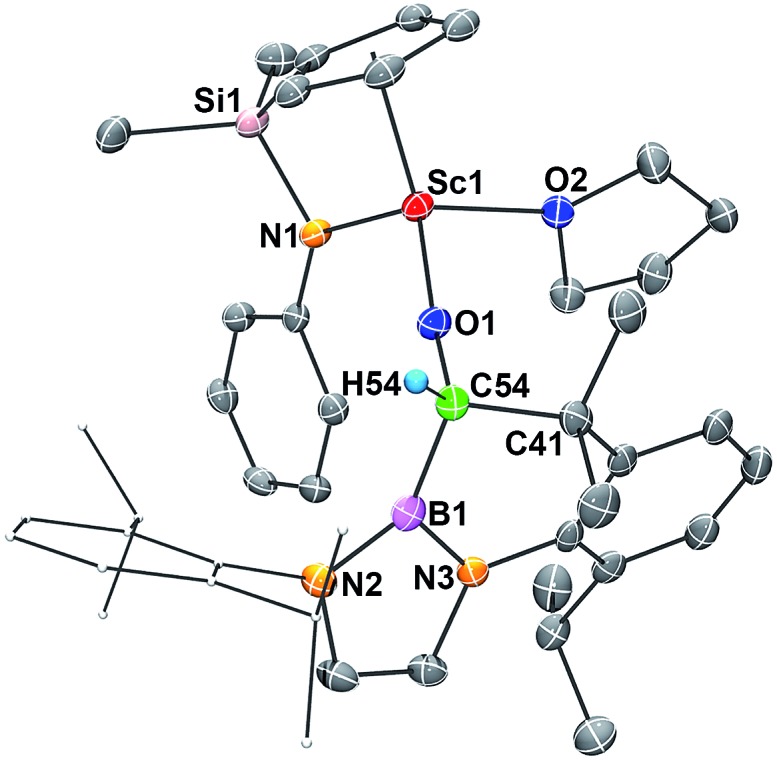
ORTEP drawing of **4** with thermal ellipsoids at the 30% level except for a 2,6-(^*i*^Pr)_2_C_6_H_3_ group in the boryl unit. Hydrogen atoms (except H54) and the Me groups on the Cp ring have been omitted for clarity. Selected bond lengths (Å) and angles (°): Sc1–O1 1.879(2), Sc1–O2 2.184(2), C54–O1 1.424(4), C41–C54 1.561(5), O1–C54 1.424(4), B1–C54 1.563(5); Sc1–O1–C54 169.5(2), B1–C54–O1 111.3(3), C41–C54–O1 110.3(3).

When complex **2** was heated at 100 °C in benzene for two days, intramolecular insertion of the carbene atom into a methine C–H bond in the boryl ligand took place to give the alkoxide complex **4** (Scheme 1). In this transformation, the Sc–carbene bond is broken, together with formation of a C54–H54 bond and a C54–C41 bond (Fig. 4). The Sc1–O1 bond distance in **4** (1.879(2) Å) is much shorter than that of the Sc–O1(oxycarbene) bond in **2** (2.114(2) Å), whilst the O1–C54 bond distance in **4** (1.424(4) Å) is much longer than that of the O1–C1(oxycarbene) bond in **2** (1.266(3) Å). The C–B bond distance in **4** (1.563(5) Å) is comparable with that in **2** (1.577(3) Å). The newly formed C54–C41 (1.561(5) Å) in **4** is best described as a single bond.

The hydrogen atom in the newly formed “HC(O)(B)C” unit in **4** gave a singlet at *δ* 3.91 in the ^1^H NMR spectrum in benzene-d_6_. The ^11^B{H} NMR signal of **4** is located at *δ* 21.8, which is comparable to that of **3** (*δ* 23.8) and is 4.9 ppm upfield shifted compared to that of **2** (*δ* 16.9). The transformation of **2** to **4** could be viewed as a typical reaction (C–H insertion) of a carbene species.^16^

The reaction of **2** with two equivalents of pyridine in benzene-d_6_ at room temperature yielded **5** as colourless crystals following recrystallization from hexane–benzene (Scheme 1; also see Fig. S1 in ESI†). In this reaction, the insertion of the carbene atom of **2** into an *ortho*-C–H bond of one molecule of pyridine took place, while another molecule of pyridine displaced the THF ligand of **2**. The ^1^H NMR spectrum of the newly formed “HC(O)(Py)B” fragment in **5** showed a singlet at *δ* 5.75 in benzene-d_6_. The ^11^B{H} NMR spectrum of **5** showed a broad peak at *δ* 25.1 which is close to that of **3** (*δ* 23.8).

**Fig. 5:**
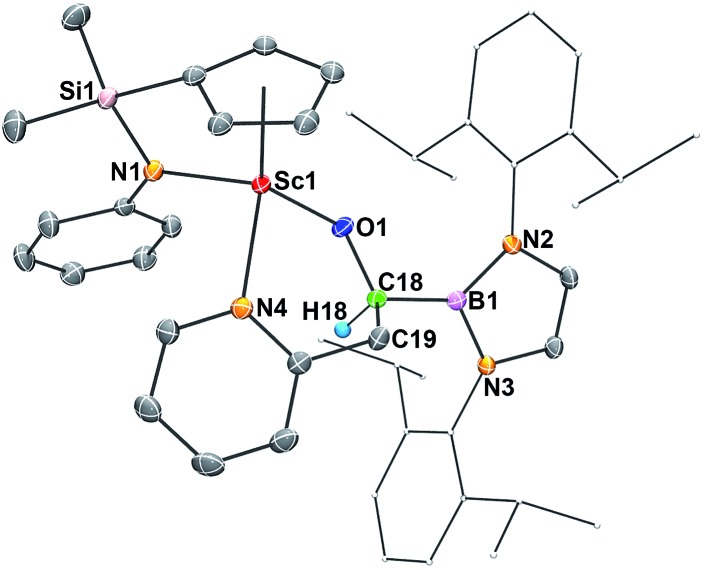
ORTEP drawing of **6** with thermal ellipsoids at the 30% level except for a 2,6-(^*i*^Pr)_2_C_6_H_3_ group in the boryl unit. Hydrogen atoms (except H18) and the Me groups on the Cp ring have been omitted for clarity. Selected bond lengths (Å): Sc1–O1 1.9175(12), Sc1–N1 2.0947(15), Sc1–N4 2.2811(15), O1–C18 1.412(2), C18–C19 1.550(2), B1–C18 1.595(3).

When **2** was allowed to react with 2-methylpyridine in benzene-d_6_ at room temperature for 26 h, the insertion of a sp^3^ C–H bond in the methyl group of 2-methylpyridine occurred to give complex **6** in 61% isolated yield (Scheme 1 and Fig. 5). The ^1^H NMR signals of the two protons on the resulting O–CH(B)–CH_2_C_5_H_4_N moiety appeared at *δ* 2.74 (dd, 10.6 Hz, 14.6 Hz) and *δ* 2.95 (d, 14.6 Hz), whilst the one of O–CH(B)–CH_2_C_5_H_4_N appeared at *δ* 4.50 (d, 10.6 Hz).

The molecular structures of **5** and **6** were also confirmed by X-ray crystallographic studies (Fig. S1† for **5** and Fig. 5 for **6**), although there were disorder problems in the case of **5**. The present C–H bond activation of pyridines by **2** is in contrast with what was observed previously in the reaction of conventional free carbene species with pyridines, in which a stable carbene–pyridine ylide complex was usually formed.^17^ The reason for the formation of the C–H activation products **5** and **6** is possibly because of facile coordination of the nitrogen atom of a pyridine unit to the electropositive Sc^3+^ centre, which could easily lead to activation of an *ortho*-C(sp^2^)–H or methyl C(sp^3^)–H bond by the highly active carbene species.^18,5g^ *ortho*-C–H activation of pyridine by a tantalum η^2^-acyl complex was reported previously.^6*a*^

### Cyclopropanation of carbene with ethylene

**Fig. 6:**
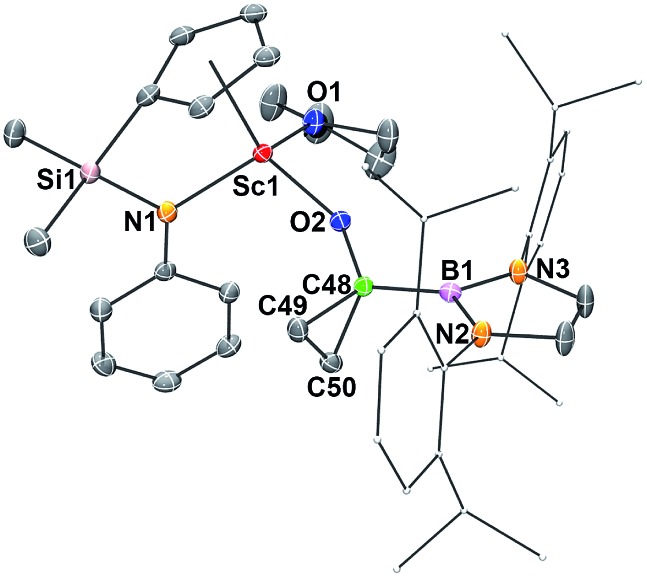
ORTEP drawing of **7** with thermal ellipsoids at the 30% level except for the 2,6-(^*i*^Pr)_2_C_6_H_3_ groups in the boryl unit. Hydrogen atoms and the Me groups on the Cp ring have been omitted for clarity. Selected bond lengths (Å) and angles (°): Sc1–N1 2.1238(18), Sc1–O1 2.1577(15), Sc1–O2 1.9083(14), O2–C48 1.402(2), C48–C49 1.514(3), C48–C50 1.533(3), C49–C50 1.492(3), C48–B1 1.569(3); C49–C48–C50 58.64(14), C50–C49–C48 61.33(14), C49–C50–C48 60.02(14).

The reaction of **2** with ethylene (1 atm) in benzene-d_6_ took place rapidly at room temperature, which was accompanied by a colour change from dark blue to colourless to give a borylcyclopropyloxy product **7** *via* the cycloaddition of the carbene atom to ethylene (Scheme 1). The Sc–O_CO_ bond distance in **7** (1.9083(14) Å) (Fig. 6) is comparable with that in **6** (1.9175(12) Å), as are the C–O_CO_ bond distances (**7**: 1.402(2) Å; **6**: 1.412(2) Å). The bond distances and angles of the triangular carbon skeleton in **6** are typical for a cyclopropyl unit (Fig. 6).

The present formation of **7** represents a rare example of cyclopropanation of ethylene with a carbene species.^19^ It was known that carbenes could undergo cyclopropanation reactions with alkenes bearing polar substituents (either electron withdrawing or donating) but are usually inert towards simple alkenes such as ethylene.^19^ The cyclopropanation of ethylene with **2** may be promoted by coordination of ethylene to the electropositive Sc^3+^ center.^20^ The cyclopropanation of a cationic iron carbene complex [{Cp(CO)_2_Fe=CHC_6_H_5_}^+^{PF_6_}^–^] with ethylene was reported previously.^19*a*^ The reaction of a classical acyl species M–C(=O)R with ethylene usually gave a straightforward insertion product formulated as [M–CH_2_CH_2_C(=O)R].^21^

## Conclusions

We have demonstrated that the reaction of a half-sandwich scandium boryl complex such as **1** with CO (1 atm) can afford a structurally characterizable oxycarbene complex including **2**, which represents the first example of a well-defined boryl-substituted oxycarbene species. The scandium boryl oxycarbene complex **2** showed diverse reactivity, such as coupling with CO to form an enediolate complex **3**, intramolecular C–H bond activation to give **4**, insertion of the carbene atom into an *ortho*-C–H bond of pyridine or into a methyl C–H bond of 2-methylpyridine, and cyclopropanation with ethylene. The structure and reactivity of the carbene species in **2** are clearly affected by the scandium ion as well as the boryl substituent. Studies on the synthesis and reactions of other rare earth metal boryl complexes are in progress.
