# Supplementary material for: Isolation, structure and reactivity of a scandium boryl oxycarbene complex
Source: Chem Sci. 2015 Sep 16;7(1):803–9. doi: 10.1039/c5sc03138a (PMC5580044; doi:10.1039/c5sc03138a)
Supplement: Supplementary file 1 [file SC-007-C5SC03138A-s001.pdf]

Electronic Supplementary Material (ESI) for Chemical Science.
This journal is © The Royal Society of Chemistry 2015

Supporting Information

Isolation, structure and reactivity of a scandium boryl oxycarbene complex

Baoli Wang,[a] Xiaohui Kang,[a,b] Masayoshi Nishiura,[a] Yi Luo*[b] and Zhaomin Hou*[a,b]

[a] Organometallic Chemistry Laboratory and RIKEN Center for Sustainable Resource Science, RIKEN, 2-1 Hirosawa, Wako, Saitama 351-0198, Japan. E-mail: houz@riken.jp

[b] State Key Laboratory of Fine Chemicals, School of Pharmaceutical Science and Technology, Dalian University of Technology, Dalian 116024, China. luoyi@dlut.edu.cn

Contents

Experimental Section S3

Synthesis and Characterization of Scandium Boryl Carbene Complex (**2**) S4

Synthesis and Characterization of Phenylamido Enolate Complex (**3**) S4

^{13}C-Labeled Experiments for Complex **3** S5

Synthesis and Characterization of Scandium Alkoxide Complex (**4**) S5

Synthesis and Characterization of Scandium Pyridyl Complex (**5**) S6

Figure S1. ORTEP drawings of **5** S6

Synthesis and Characterization of Scandium Methyl-Pyridyl Complex (**6**) S7

Synthesis and Characterization of Scandium Cyclopropyl Complex (**7**) S7

Figure S2. ^{13}C NMR spectrum of **2** S8

Figure S3. ^{13}C NMR spectrum of **2-^{13}C** S8

Figure S4. ^{13}C NMR spectrum of **3** S8

Figure S5. ^{13}C NMR spectrum of **3-^{13}C$_2$** S9

Table S1. Crystal data and structure refinement for complex **5** S10

Table S2. Atomic coordinates and equivalent isotropic displacement parameters for complex **5** S11

Table S3. Bond lengths and angles for complex **5** S13

Table S4. Anisotropic displacement parameters for complex **5** S27

Computational Section S30

Figure S7. Optimized structure of complex **2** S30

Table S5. Structural comparison between X-ray structure and DFT optimized structure of complex **2** S30

Table S6. Selected calculated and experimental IR data for **2** and **2-^{13}C** S31

Figure S8. Computational IR spectrum of **2** S31

Figure S9. Computational IR spectrum of **2-^{13}C** S31

Table S7. Second order perturbation theory analysis for **2** S32

References S33

Experimental Section

General Information

All manipulations of air- and moisture-sensitive compounds were performed under an argon atmosphere using standard Schlenk techniques or a nitrogen atmosphere in an MBRAUN Labmaster 130 glovebox. Argon and nitrogen (Takachiho Chemical Industrial Co., Ltd.) were purified by passing through a Dryclean column (4 A molecular sieves, Nikka Seiko Co.) and a Gasclean GC-XR column (Nikka Seiko Co.). The oxygen and moisture concentrations in the glovebox atmosphere were monitored by an O_2/H_2O Combi–Analyzer (MBRAUN) to ensure both were always below 0.1 ppm. Elemental analyses were performed by a MICRO CORDER JM10. Organic solvents were obtained from Kanto Kagaku Co., purified by an MBRAUN SPS-800 Solvent Purification System and dried over fresh Na chips in a glovebox. Carbon monoxide and ^{13}C-enriched carbon monoxide (99 atom%, ISOTEC) were used as received without further purification. Half-sandwich scandium boryl complex **1** was synthesized according to the literature method.[1] Pyridine and methylpyridine were purchased from TCI, dried over CaH_2, vacuum-transferred, degassed by two freeze-pump-thaw cycles and kept in the glovebox. All ^{1}H, ^{13}C and ^{11}B NMR spectra of complexes were recorded on an ECS-400 instrument in C_6D_6 with tetramethylsilane (TMS) as an internal standard, unless otherwise mentioned. Data are reported as follows: chemical shift in ppm (δ), multiplicity (s = singlet, d = doublet, sep = septet, m = multiplet, br = broad signal), coupling constant (Hz), integration. Infrared spectra were recorded on a Nicolet 380 (Thermo Electron) spectrometer using nujol mulls between KBr plates.

X-ray Crystallographic Studies

A crystal was sealed in a thin-walled glass capillary under a microscope in the glove box. Data collections were performed at –100 ˚C on a Bruker SMART APEX diffractometer with a CCD area detector using graphite-monochromated MoK_α radiation (λ= 0.71069 Å). The determination of crystal class and unit cell parameters was carried out using the SMART program package.[2] The raw frame data were processed using SAINT[3] and SADABS[4] to yield the reflection data file. The structures were solved using the SHELXTL program.[5] Refinements were performed on F^2 anisotropically for all the non-hydrogen atoms by the full-matrix least-squares method. The analytical scattering factors for neutral atoms were used throughout the analysis using the SHELXTL program. The hydrogen atoms were placed at the calculated positions and were included in the structure calculation without further refinement of their parameters. The residual electron densities were of no chemical significance.

CCDC numbers CCDC 981561 (**2**), 981563 (**3**), 981562 (**4**), 1042268 (**6**), and 1042269 (**7**) contain the supplementary crystallographic data for this paper. These data can be obtained free of charge from The Cambridge Crystallographic Data Centre via www.ccdc.cam.uk/data_request/cif

Synthesis and Characterization of Scandium Boryl Carbene Complex (2)

A benzene solution (5 mL) of **1** (0.527 g, 0.549 mmol) in a 10-mL Schlenk tube was frozen in liquid nitrogen, evacuated under vacuum and then backfilled with carbon monoxide (1 atm). The reaction solution was warmed to room temperature and stirred for 5 min. Then volatiles were removed under reduced pressure, and the residue was extracted with hexane. Dark blue crystals of **2** (0.383 g, 87%) were grown from a concentrated hexane solution at –30 °C. ^{1}H NMR (400 MHz, C_6D_6, 25 °C): δ 0.86 (s, 3H, Si(*Me*)$_2$), 0.88 (s, 3H, Si(*Me*)$_2$), 0.98–1.01 (m, 4H, THF), 1.16–1.22 (m, 24H, CH(*Me*)$_2$), 1.52 (s, 3H, Cp(*Me*)$_4$), 1.61 (s, 3H, Cp(*Me*)$_4$), 1.93 (s, 3H, Cp(*Me*)$_4$), 2.11 (s, 3H, Cp(*Me*)$_4$), 2.97 (sep, 6.9 Hz, 2H, *CH*(Me)$_2$), (3.23 (sep, 7.0 Hz), 3.36–3.41 (m), 2H, *CH*(Me)$_2$), 3.47–3.54 (m, 4H, THF), 6.17 (s, 2H, N–C*H*=C*H*–N), 6.29–6.31 (m, 2H, Aryl), 6.70–6.73 (m, 1H, Aryl), 7.07–7.24 (m, 8H, Aryl); ^{13}C NMR (100 MHz, C_6D_6, 25 °C, δ_{C6D6} = 128.06): δ 3.7, 5.2 (s, 2C, Si(*Me*)$_2$), 10.9, 11.8, 13.3, 14.0 (s, 4C, Cp(*Me*)$_4$), 22.7, 24.2, 25.1, 25.6 (s, 8C, CH(*Me*)$_2$), 25.1 (s, 2C, THF), 28.4, 29.4 (s. 2C, *C*H(Me)$_2$), 71.4 (s, 2C, THF), 119.3 (s, 2C, N–*C*H=*C*H–N), 107.6, 115.7, 120.6, 121.8, 123.3, 123,5, 123.6, 124.7, 126.7, 127.6, 128.9, 129.2, 139.5, 145.5, 146.4, 155.7 (aromatic and Cp ring carbons), 427.4 (s, 1C, Sc–C_{CO}). (See Figure S1 for ^{13}C NMR spectrum). ^{11}B NMR (160 MHz, C_6D_6, 25 °C): δ 16.9 (br). Anal. Calcd. for $C_{48}H_{67}BN_3O_2ScSi$: C 71.89; H 8.42; N 5.24. Found: C 72.34; H 8.68; N 5.14.

Complex **2-^{13}C** was obtained via an analogous method using ^{13}C-enriched CO. The NMR data were found to be comparable to **2**. (See Figures S2 and S3 for ^{13}C NMR spectrum).

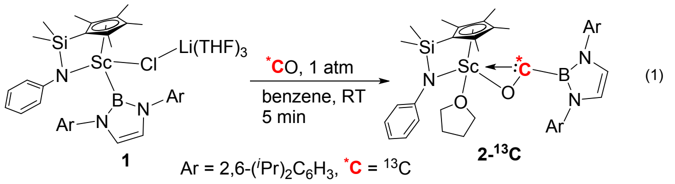

Scheme S1 Synthesis of ^{13}C labeled complex **2** (**C***: ^{13}C)

Synthesis and Characterization of Phenylamido Enolate Complex (3)

A benzene/THF solution (5 mL/1 mL) of **2** (0.329 g, 0.410 mmol) in a 10-mL Schlenk tube was frozen in liquid nitrogen, evacuated under vacuum and then backfilled with carbon monoxide (1 atm). The reaction solution was slowly warmed to room temperature and stirred for 3 min. Then CO and solvent was removed under reduced pressure, and the residue was extracted with hexane. Yellow crystals of **3** (0.300 g, 81%) were grown from a concentrated hexane solution at –30 °C. ^{1}H NMR (400 MHz, C_6D_6, 25 °C): δ 0.32 (s, 3H, Si(*Me*)$_2$), 0.51 (s, 3H, Si(*Me*)$_2$), 1.21–1.29 (m, 18H, CH(*Me*)$_2$, 8H, THF), 1.49 (d, 5.5 Hz, 6H, CH(*Me*)$_2$), 1.57 (s, 3H, Cp(*Me*)$_4$), 1.74 (s, 3H, Cp(*Me*)$_4$), 1.81 (s, 3H, Cp(*Me*)$_4$), 2.27 (s, 3H, Cp(*Me*)$_4$), 3.19–3.35 (m, 2H, *CH*(Me)$_2$), 3.48 (br, 8H, THF), 3.94 (br, 2H, *CH*(Me)$_2$), 5.96 (br, 2H, Aryl), 6.40 (s, 2H, N–C*H*=C*H*–N), 6.75 (t, 6.7 Hz, 1H, Aryl), 7.15–7.19 (m, 4H, Aryl), 7.35 (d, 4.6 Hz, 4H, Aryl); ^{11}B NMR (160 MHz, C_6D_6, 25 °C): δ 23.8 (br). (See Figure S3 for ^{13}C NMR spectrum of **3**) Anal. Calcd. for $C_{53}H_{75}BN_3O_4ScSi$: C 70.57; H 8.38; N 4.66. Found: C 70.85; H 8.29; N 4.49.

Synthesis and Characterization of ^{13}C-labled Phenylamido Enolate (3-$^{13}C_2$)

Complex **3-$^{13}C_2$** was obtained via an analogous method from the reaction of **2-^{13}C** with ^{13}C-enriched CO. ^{13}C NMR (100 MHz, C_6D_6, 25 °C, δ_{C6D6} = 128.06): δ 3.4, 3.8 (s, 2C, Si(*Me*)$_2$), 10.3,

11.0, 15.1, 15.5 (s, 4C, Cp(*Me*)$_4$), 23.4 (s, 4C, THF), 23.6, 25.4, 26.7, 26.9 (s, 8C, CH(*Me*)$_2$), 28.6, 28.9 (s, 4C, *C*H(Me)$_2$), 69.9 (s, 4C, THF), 121.1 (s, 2C, N–*C*H=*C*H–N), 114.3, 116.3, 118.7, 119.8, 123.2, 123.8, 127.0, 128.6, 130.9, 136.4, 141.4, 146.5, 147.3, 150.3 (aromatic and Cp ring carbons), 134.6 (br, d, 70.2 Hz, Sc–O–^{13}*C*–B), 136.7 (d, 76.4 Hz, 1C, Si–O–^{13}*C*). (See Figure S4 for ^{13}C NMR spectrum of **3-^{13}C$_2$**)

^{13}C-Labeled Experiments for Complex 3

Complex **2** reacted with ^{13}C-enriched CO to give complex **3-^{13}C** (Eq. 2). In the ^{13}C NMR spectrum, a sharp peak at δ 136.7 was observed. On the other hand, the reaction of ^{13}C labeled complex **2-^{13}C** with CO produced another complex **3-^{13}C'**. ^{13}C NMR spectrum showed a broad peak at δ 134.7, suggesting that the ^{13}C atom linked to the boryl unit because of ^{13}C-^{11}B coupling. The ^{13}C NMR spectrum of **3-^{13}C$_2$** showed two doublets at δ 134.7 and 136.7, suggesting the presence of a ^{13}C=^{13}C unit, and both C atoms in the OCCO unit originate from external CO molecules.

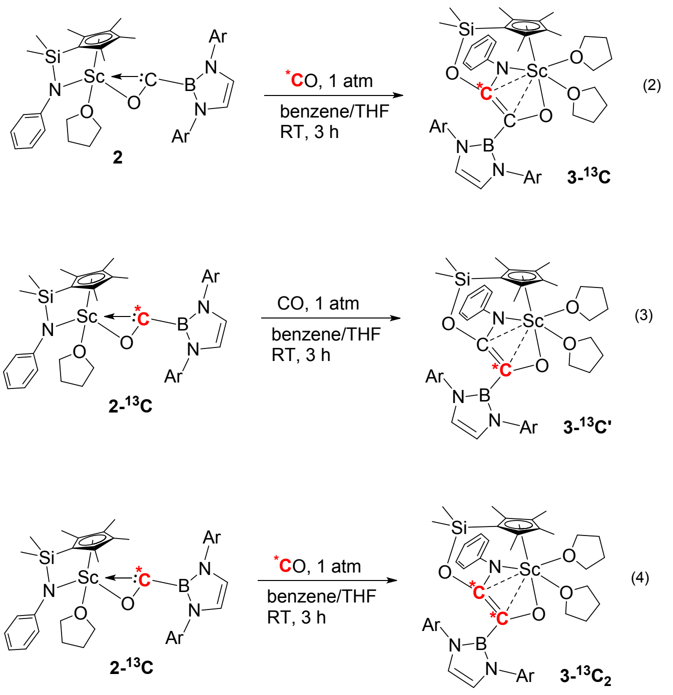

Scheme S2 Synthesis of three types of ^{13}C labeled complex **3** (Ar = 2,6-(^{i}Pr)$_2$C$_6$H$_3$, **C***: ^{13}C)

Synthesis and Characterization of Scandium Alkoxide Complex (4)

A benzene solution (5 mL) of **2** (0.570 g, 0.711 mmol) in a 10-mL Schlenk tube was heated at 100 °C for 2 days. Solvent was removed under reduced pressure, and the residue was extracted with hexane. Colourless crystals of **4** (0.239 g, 42%) were grown from a concentrated hexane solution at –30 °C. ^{1}H NMR (400 MHz, C$_6$D$_6$, 25 °C): δ 0.79 (s, 3H, Si(*Me*)$_2$), 0.77–0.82 (m, 4H, THF), 0.90 (s, 3H, Si(*Me*)$_2$), 1.00 (d, 6.4 Hz, 3H, CH(*Me*)$_2$), 1.05–1.07 (m, 6H, CH(*Me*)$_2$), 1.13–1.16 (m, 6H, CH(*Me*)$_2$, –C(*Me*)$_2$), 1.27 (d, 6.9 Hz, 3H, CH(*Me*)$_2$), 1.38 (d, 6.8 Hz, 3H, CH(*Me*)$_2$), 1.87 (s, 3H, –C(*Me*)$_2$), 1.88 (s, 3H, Cp(*Me*)$_4$), 2.000 (s, 3H, Cp(*Me*)$_4$), 2.003 (s, 3H, Cp(*Me*)$_4$), 2.04 (s, 3H, Cp(*Me*)$_4$), 3.01–3.05 (m, 4H, THF), 3.14 (sep, 6.9 Hz, 1H, *CH*(Me)$_2$), 3.21 (sep, 6.9 Hz, 1H, *CH*(Me)$_2$), 3.91 (s, 1H, Sc–O–C*H*), 4.05 (sep, 6.8 Hz, 1H, *CH*(Me)$_2$), 6.19 (d, 2.3 Hz, 1H,

N–CH=CH–N), 6.72 (t, 7.1 Hz, 1H, Aryl), 6.81–6.83 (m, 2H, Aryl), 6.89 (d, 2.3 Hz, 1H, N–CH=CH–N), 6.70–7.04 (m, 2H, Aryl), 7.12–7.21 (m, 6H, Aryl); ^{13}C NMR (100 MHz, C_6D_6, 25 °C): δ 3.48, 5.10 (s, 2C, Si(Me)$_2$), 11.30, 11.73, 13.68, 13.95 (s, 4C, Cp(Me)$_4$), 24.07, 24.30, 25.14, 25.41, 25.87, 25.95, 26.00, 26.41 (s, 8C, CH(Me)$_2$, –C(Me)$_2$), 24.89 (s, 2C, THF), 43.50 (s, 1C, –C(Me)$_2$), 71.73 (s, 2C, THF), 73.13 (br, 1C, Sc–O–CH), 114.87, 121.26 (s, 2C, N–CH=CH–N), 107.19, 115.78, 120.34, 121.41, 122.87, 123.46, 123.88, 124.55, 125.47, 126.12, 126,35, 129.38, 129.61, 137.89, 138.31, 138.39, 139.89, 145.81, 147.86, 154.80 (aromatic and Cp ring carbons). ^{11}B NMR (160 MHz, C_6D_6, 25 °C): δ 21.8 (br). Anal. Calcd. for $C_{48}H_{67}BN_3O_2ScSi$: C 71.89; H 8.42; N 5.24. Found: C 72.36; H 8.33; N 5.69.

Synthesis and Characterization of Scandium Pyridyl Complex (5)

A hexane solution (5 mL) of pyridine (0.013 g, 0.16 mmol) was added into a benzene solution (5 mL) of **2** (0.066 g, 0.082 mmol) at room temperature and the mixture was stirred for 0.5 hour. After solvent removal under reduced pressure, the residue was extracted with hexane. The filtrate was further concentrated to afford colourless crystals of **5** (0.056 g, 76%) at –30 ˚C. ^{1}H NMR (400 MHz, C_6D_6, 25 ˚C): δ 0.79 (d, 6.4 Hz, 6H, CH(Me)$_2$), 0.98 (s, 3H, Si(Me)$_2$), 1.05 (s, 3H, Si(Me)$_2$), 1.09 (d, 6.4 Hz, 6H, CH(Me)$_2$), 1.27 (d, 6.4 Hz, 6H, CH(Me)$_2$), 1.35 (d, 6.4 Hz, 6H, CH(Me)$_2$), 1.43 (s, 3H, Cp(Me)$_4$), 1.45 (s, 3H, Cp(Me)$_4$), 1.71 (s, 3H, Cp(Me)$_4$), 1.84 (s, 3H, Cp(Me)$_4$), 3.28 (sep, 6.8 Hz, 2H, CH(Me)$_2$), 3.59 (sep, 6.8 Hz, 2H, CH(Me)$_2$), 5.75 (s, 1H, O–CH–B), 6.09 (t, 6.2 Hz, 1H, Py), 6.27 (s, 2H, N–CH=CH–N), 6.52–6.59 (m, 4H, Py and Aryl), 6.76 (t, 7.1 Hz, 1H, Aryl), 6.86 (t, 7.6 Hz, 1H, Py), 6.97–7.01 (m, 1H, Py), 7.06–7.21 (m, 9H, Py and Aryl), 8.31 (d, 5.0 Hz, 1H, Py), 8.92 (d, 5.0 Hz, 2H, Py). ^{13}C NMR (100 MHz, C_6D_6, 25 ˚C, δ_{C6D6} = 128.06 ppm): δ 4.4, 4.6 (s, 2C, Si(Me)$_2$), 11.1, 11.9, 13.8, 14.8 (s, 4C, Cp(Me)$_4$), 22.3, 24.1, 25.3, 26.6 (s, 8C, CH(Me)$_2$) 28.5, 28.7 (s. 4C, CH(Me)$_2$), 70.7 (br, 1C, O–CH–B), 120.2 (s, 2C, N–CH=CH–N), 106.6, 118.9, 120.1, 123.9, 137.2, 138.2, 150.4, 151.5, 175.2 (Pyridine carbons), 106.6, 115.2, 120.5, 123.5, 123.7, 125.1, 126.4, 126.5, 128.6, 129.7, 139.5, 145.8, 146.0, 146.8, 155.3 (aromatics and Cp ring carbons). ^{11}B NMR (160 MHz, C_6D_6, 25 ˚C): δ 25.1 (br). Anal. Calcd. for $C_{54}H_{69}BN_5OScSi$: C 73.04; H 7.83; N 7.89. Found: C 73.20; H 8.00; N 7.58.

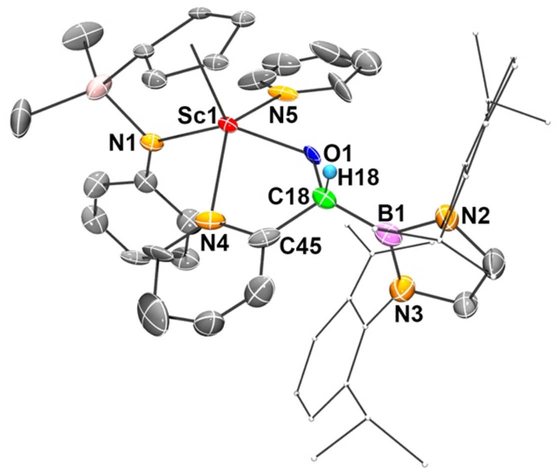

Figure S1 ORTEP drawings of **5** with thermal ellipsoids at the 30% level except for a 2,6-(^{i}Pr)$_2$C$_6$H$_3$ group in the boryl unit. Hydrogen atoms (except H18) and the Me groups on the Cp ring have been omitted for clarity. Selected bond lengths (Å) and angles (deg): Sc1–O1 1.990(4), Sc1–N1 2.173(7), Sc1–N4 2.297(10), Sc1–N5 2.321(9), O1–C18 1.415(12), C18–C45 1.526(16),

B1–C18 1.570(17).

Synthesis and Characterization of Scandium Methyl-Pyridyl Complex (6)

A hexane solution (5 mL) of 2-methylpyridine (0.011 g, 0.12 mmol) was added to a benzene solution (5 mL) of **2** (0.095 g, 0.12 mmol) at room temperature and the mixture was stirred for 26 hour. After solvent removal under reduced pressure, the residue was dissolved with hexane and keep at –30 ˚C to give colourless crystals of **6** (0.059 g, 61%) at –30 ˚C. ^{1}H NMR (400 MHz, C_6D_6, 25 ˚C): δ 0.92 (s, 3H, Si(*Me*)$_2$), 0.99 (s, 3H, Si(*Me*)$_2$), 1.18 (d, 6.8 Hz, 6H, CH(*Me*)$_2$), 1.20 (d, 6.8 Hz, 6H, CH(*Me*)$_2$), 1.32 (d, 6.8 Hz, 6H, CH(*Me*)$_2$), 1.33 (d, 6.8 Hz, 6H, CH(*Me*)$_2$), 1.48 (s, 3H, Cp(*Me*)$_4$), 1.65 (s, 3H, Cp(*Me*)$_4$), 2.01 (s, 3H, Cp(*Me*)$_4$), 2.23 (s, 3H, Cp(*Me*)$_4$), 2.74 (dd, 1H, 10.6 Hz, 14.6 Hz, Py–C*H*$_2$), 2.95 (d, 1H, 14.6 Hz, Py–C*H*$_2$), 3.25 (sep, 7.0 Hz, 2H, *CH*(Me)$_2$), 3.37 (sep, 7.0 Hz, 2H, *CH*(Me)$_2$), 4.50 (d, 10.6 Hz, 1H, O–C*H*–B), 6.00 (t, 6.2 Hz, 1H, Py), 6.12 (s, 2H, N–C*H*=C*H*–N), 6.34 (d, 7.8 Hz, 1H, Py), 6.57–6.61 (m, 1H, Py), 6.66–6.68 (m, 2H, Ar), 6.72–6.76 (m, 1H, Ar), 7.08–7.26 (m, 8H, Ar), 8.00 (d, 5.5 Hz, 1H, Py). ^{13}C NMR (100 MHz, C_6D_6, 25 ˚C, δ_{C6D6} = 128.06 ppm): δ 4.0, 5.2 (s, 2C, Si(*Me*)$_2$), 10.9, 11.8, 14.4, 14.7 (s, 4C, Cp(*Me*)$_4$), 23.1, 24.1, 25.4, 25.9 (s, 8C, CH(*Me*)$_2$) 28.7, 28.9 (s. 4C, *C*H(Me)$_2$), 47.8 (br, 1C, Py–*C*H$_2$), 68.9 (br, 1C, O–*C*H–B), 120.0 (s, 2C, N–*C*H=*C*H–N), 121.7, 124.3, 139.7, 148.7, 163.3 (Pyridine carbons), 109.1, 116.4, 120.1, 123.6, 123.9, 124.1, 125.6, 126.6, 127.6, 128.6, 129.3, 139.8, 145.7, 146.5, 155.1 (aromatics and Cp ring carbons). ^{11}B NMR (160 MHz, C_6D_6, 25 ˚C): δ 25.3 (br). Anal. Calcd. for $C_{50}H_{66}BN_4OScSi$: C 72.97; H 8.08; N 6.81. Found: C 73.19; H 8.36; N 6.64.

Synthesis and Characterization of Scandium Cyclopropyl Complex (7)

A benzene solution (5 mL) of **2** (0.361 g, 0.450 mmol) in a 50-mL schlenk tube was frozen in liquid nitrogen, evacuated under vacuum and then backfilled with ethylene (1 atm). The reaction solution was slowly warmed to room temperature and stirred for 10 min. Then ethylene and solvent was removed under reduced pressure, and the residue was dissolved with hexane and kept at –30 ˚C to give colorless crystals **7** (0.317g, 85%). ^{1}H NMR (400 MHz, C_6D_6, 25 ˚C): δ 0.30–0.34 (m, 1H, cyclic C*H*$_2$), 0.48–0.54 (m, 2H, cyclic C*H*$_2$), 0.72–0.78 (m, 1H, cyclic C*H*$_2$), 0.80, 0.81 (s, 6H, Si(*Me*)$_2$), 1.01–1.05 (m, 4H, THF), 1.19–1.25 (m, 18H, CH(*Me*)$_2$), 1.41 (d, 6.9 Hz, 6H, CH(*Me*)$_2$), 1.77 (s, 3H, Cp(*Me*)$_4$), 1.981 (s, 3H, Cp(*Me*)$_4$), 2.13 (s, 3H, Cp(*Me*)$_4$), 2.18 (s, 3H, Cp(*Me*)$_4$), , 2.88–2.94 (m, 2H, THF), 3.15–3.20 (m, 2H, THF), 3.26 (sep 6.9 Hz, 2H, *CH*(Me)$_2$), 3.53 (sep, 6.9 Hz, 2H, *CH*(Me)$_2$), 5.97 (s, 2H, N–C*H*=C*H*–N), 6.78 (t, 7.3 Hz, 1H, Aryl), 6.87 (d, 7.3 Hz, 2H, Aryl), 7.05–7.25 (m, 8H, Aryl). ^{11}B NMR (160 MHz, C_6D_6, 25 ˚C): δ 24.3 (br). Anal. Calcd. for $C_{48}H_{67}BN_3O_2ScSi$: C 72.36; H 8.62; N 5.06. Found: C 72.12; H 8.76; N 4.59. Following the procedure of synthesis of **7**, **7-^{13}C** was obtained in 81% yield through the reaction of **2-^{13}C** with ethylene. The ^{1}H NMR data of **7-^{13}C** was similar to that of **7**. ^{13}C NMR (100 MHz, C_6D_6, 25 ˚C, δ_{C6D6} = 128.06 ppm): δ 4.0, 4.8 (s, 2C, Si(*Me*)$_2$), 11.3, 12.1, 14.3, 14.8 (s, 4C, Cp(*Me*)$_4$), 17.9, 18.0, 18.7, 18.8 (s, 2C, cyclic *C*H$_2$), 25.4 (s, 2C, THF), 23.7, 24.1, 25.5, 26.7 (s, 8C, CH(*Me*)$_2$), 28.5, 28.8 (s, 4C, *C*H(Me)$_2$), 50.4 (br, O–*C*–B), 71.7 (s, 2C, THF), 120.3 (s, 2C, N–*C*H=*C*H–N), 107.7, 116.5, 120.8, 122.3, 123.3, 123.8, 125.6, 127.3, 128.1, 129.1, 141.3, 146.2, 146.4, 154.8 (aromatics and Cp ring carbons).

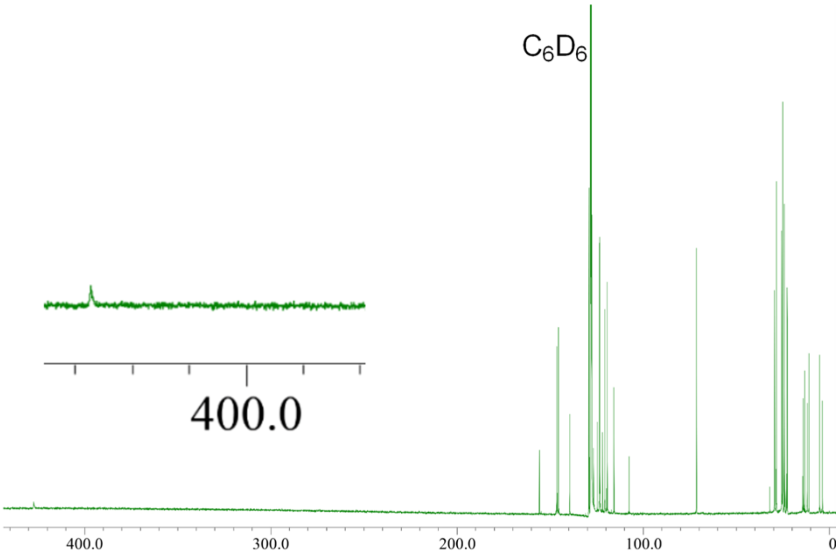

Figure S2. ^{13}C NMR spectrum of **2**

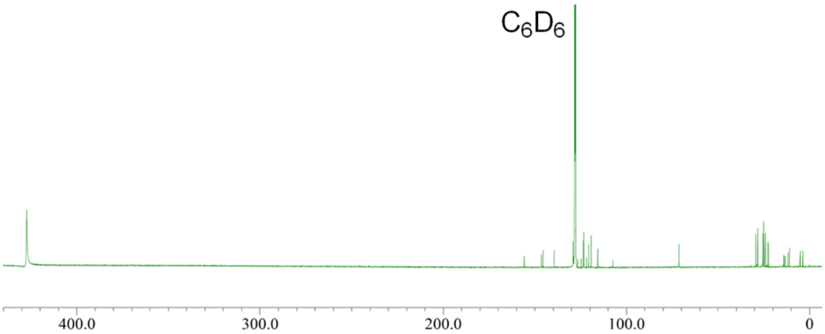

Figure S3. ^{13}C NMR spectrum of **2-^{13}C**

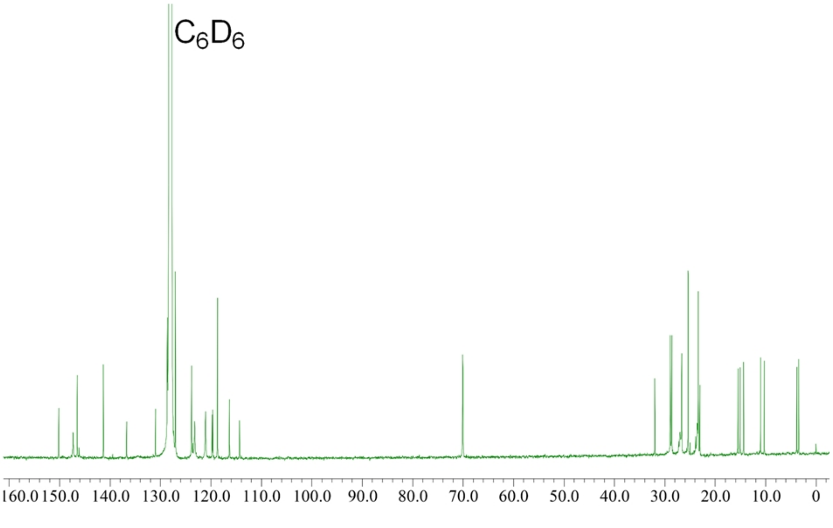

Figure S4. ^{13}C NMR spectrum of **3**

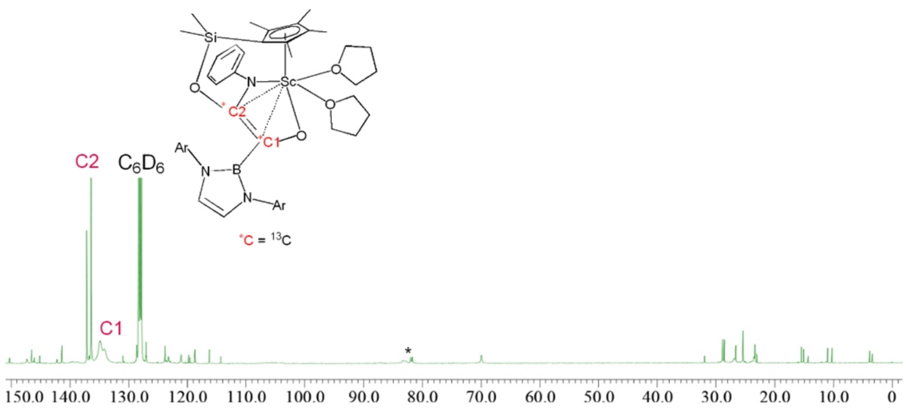

Figure S5. ^{13}C NMR spectrum of **3-^{13}C$_2$** (*: impurity)

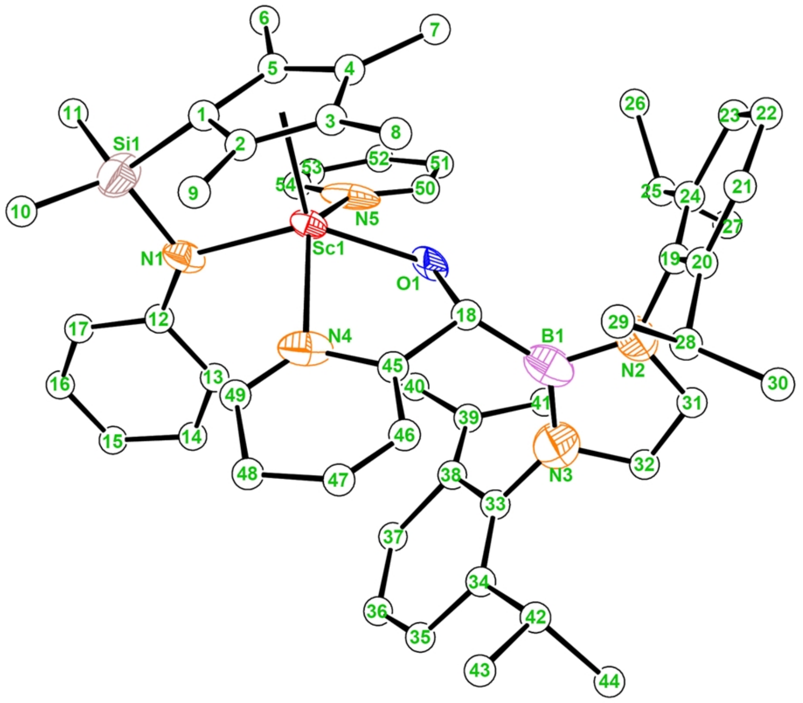

Figure S6 ORTEP drawings of **5** with thermal ellipsoids at the 30% level. Hydrogen atoms have been omitted for clarity

Table S1. Crystal data and structure refinement for complex **5**.

Identification code	complex **5**	
Empirical formula	C63 H78 B N5 O Sc Si	
Formula weight	1005.16	
Temperature	173(2) K	
Wavelength	0.71073 Å	
Crystal system	Monoclinic	
Space group	$P2_1$	
Unit cell dimensions	a = 11.2472(10) Å	α = 90°.
	b = 16.3797(16) Å	β = 97.593(4)°.
	c = 17.0307(16) Å	γ = 90°.
Volume	3110.0(5) Å^3	
Z	2	
Density (calculated)	1.073 Mg/m^3	
Absorption coefficient	0.179 mm^{-1}	
F(000)	1078	
Crystal size	0.300 x 0.200 x 0.200 mm^3	
Theta range for data collection	1.206 to 24.997°.	
Index ranges	-13<=h<=13, -19<=k<=19, -20<=l<=20	
Reflections collected	67586	
Independent reflections	10941 [R(int) = 0.0992]	

Completeness to theta = 24.997°	100.0 %
Refinement method	Full-matrix least-squares on F^2
Data / restraints / parameters	10941 / 5 / 629
Goodness-of-fit on F^2	1.297
Final R indices [I>2sigma(I)]	R1 = 0.1163, wR2 = 0.2997
R indices (all data)	R1 = 0.1530, wR2 = 0.3330
Absolute structure parameter	0.50(2)
Largest diff. peak and hole	1.415 and -0.550 e.Å^{-3}

Table S2. Atomic coordinates (x 10^4) and equivalent isotropic displacement parameters (Å^{2}x 10^3) for complex **5**. U(eq) is defined as one third of the trace of the orthogonalized U^{ij} tensor.

	x	y	z	U(eq)
Sc(1)	6966(1)	7149(1)	9815(1)	25(1)
Si(1)	7521(2)	7219(3)	11624(1)	53(1)
B(1)	6749(10)	7775(8)	7311(8)	49(3)
O(1)	6706(5)	7141(5)	8637(3)	31(1)
N(1)	8345(6)	7219(6)	10835(4)	39(2)
N(2)	5882(8)	7596(6)	6654(5)	54(2)
N(3)	7824(9)	7892(7)	6975(5)	62(3)
N(4)	7514(8)	8435(6)	9461(6)	48(2)
N(5)	7590(8)	5838(5)	9554(7)	46(3)
C(1)	5998(7)	7205(8)	11021(5)	34(2)
C(2)	5518(9)	7844(7)	10581(7)	38(3)
C(3)	4808(8)	7558(6)	9897(6)	31(2)
C(4)	4787(8)	6694(6)	9908(6)	32(2)
C(5)	5542(8)	6448(6)	10591(6)	27(2)
C(6)	5701(10)	5582(7)	10852(7)	46(3)
C(7)	4093(10)	6157(8)	9294(7)	49(3)
C(8)	4055(11)	8083(7)	9316(8)	52(3)
C(9)	5573(12)	8751(7)	10786(9)	56(3)
C(10)	7756(15)	8155(12)	12280(8)	89(6)
C(11)	7821(12)	6344(13)	12293(10)	95(6)
C(12)	9587(7)	7200(8)	10897(5)	41(2)
C(13)	10181(7)	7181(9)	10225(6)	50(2)

C(14)	11401(8)	7173(10)	10260(7)	60(3)
C(15)	12119(7)	7183(8)	11004(6)	48(2)
C(16)	11574(8)	7168(10)	11655(7)	61(3)
C(17)	10344(8)	7237(10)	11611(6)	57(3)
C(18)	6464(9)	7861(6)	8185(6)	42(2)
C(19)	4564(15)	7450(17)	6597(8)	91(7)
C(20)	3861(15)	8053(13)	6437(9)	84(5)
C(21)	2716(13)	7859(12)	6374(9)	84(5)
C(22)	2190(11)	7128(16)	6324(8)	94(5)
C(23)	3040(30)	6413(17)	6494(12)	181(17)
C(24)	4288(16)	6673(12)	6605(9)	82(5)
C(25)	5119(17)	5909(11)	6783(9)	91(5)
C(26)	4600(20)	5228(12)	7271(10)	113(7)
C(27)	5460(20)	5568(15)	6037(14)	123(7)
C(28)	4214(13)	8955(10)	6402(8)	71(4)
C(29)	3840(20)	9449(12)	7064(13)	120(7)
C(30)	3750(30)	9370(16)	5617(10)	158(11)
C(31)	6449(11)	7615(10)	5946(7)	71(4)
C(32)	7562(10)	7815(10)	6143(7)	70(4)
C(33)	9017(12)	8101(11)	7315(10)	74(5)
C(34)	9461(10)	8870(7)	7238(7)	56(3)
C(35)	10652(10)	9049(9)	7581(7)	60(3)
C(36)	11328(11)	8458(9)	8004(7)	66(3)
C(37)	10886(11)	7697(9)	8085(8)	69(4)
C(38)	9600(20)	7484(15)	7752(9)	103(7)
C(39)	9469(17)	6667(8)	7757(9)	78(5)
C(40)	10039(14)	6071(10)	8329(10)	81(4)
C(41)	9111(13)	6207(9)	7080(14)	97(6)
C(42)	8786(13)	9533(10)	6725(9)	78(4)
C(43)	8924(15)	10398(10)	7094(15)	112(7)
C(44)	9230(20)	9580(16)	5880(10)	123(7)
C(45)	6984(10)	8594(8)	8667(7)	52(3)
C(46)	7044(14)	9369(8)	8391(8)	68(4)
C(47)	7546(16)	9912(9)	8808(9)	75(4)
C(48)	8169(18)	9862(13)	9570(13)	112(7)
C(49)	8113(13)	8986(7)	9913(10)	69(4)
C(50)	7255(11)	5485(9)	8886(10)	71(5)
C(51)	7616(15)	4600(12)	8822(12)	87(5)

C(52)	8268(18)	4328(13)	9419(16)	121(9)
C(53)	8755(15)	4622(8)	10033(12)	83(5)
C(54)	8277(14)	5393(8)	10098(11)	74(5)
C(55)	4200(30)	7880(20)	3630(20)	172(13)
C(56)	4457(19)	7580(20)	2930(11)	118(8)
C(57)	4558(16)	6758(19)	2881(14)	110(8)
C(58)	4120(20)	6295(16)	3418(15)	114(7)
C(59)	3800(20)	6620(30)	4050(20)	142(11)
C(60)	3820(20)	7380(30)	4188(15)	168(19)
C(61)	9660(40)	7430(20)	4430(20)	175(9)
C(62)	8680(30)	7940(30)	4470(20)	175(9)
C(63)	8790(30)	8780(30)	4350(30)	175(9)
C(64)	9880(40)	9100(20)	4190(20)	175(9)
C(65)	10860(30)	8590(30)	4150(20)	175(9)
C(66)	10750(30)	7760(30)	4270(20)	175(9)

Table S3. Bond lengths [Å] and angles [°] for complex **5**.

Sc(1)-O(1)	1.990(5)
Sc(1)-N(1)	2.173(7)
Sc(1)-N(4)	2.297(10)
Sc(1)-N(5)	2.321(9)
Sc(1)-C(1)	2.449(7)
Sc(1)-C(5)	2.488(9)
Sc(1)-C(2)	2.491(10)
Sc(1)-C(3)	2.540(9)
Sc(1)-C(4)	2.586(9)
Sc(1)-Si(1)	3.063(3)
Si(1)-N(1)	1.731(7)
Si(1)-C(11)	1.835(16)
Si(1)-C(1)	1.877(8)
Si(1)-C(10)	1.895(16)
B(1)-N(2)	1.415(16)
B(1)-N(3)	1.417(16)
B(1)-C(18)	1.570(17)
O(1)-C(18)	1.415(12)
N(1)-C(12)	1.387(10)
N(2)-C(31)	1.436(15)

N(2)-C(19)	1.493(17)
N(3)-C(32)	1.415(15)
N(3)-C(33)	1.432(17)
N(4)-C(49)	1.313(17)
N(4)-C(45)	1.427(15)
N(5)-C(50)	1.287(16)
N(5)-C(54)	1.340(18)
C(1)-C(2)	1.356(16)
C(1)-C(5)	1.496(15)
C(2)-C(3)	1.403(16)
C(2)-C(9)	1.527(15)
C(3)-C(4)	1.415(11)
C(3)-C(8)	1.488(16)
C(4)-C(5)	1.405(14)
C(4)-C(7)	1.503(15)
C(5)-C(6)	1.490(15)
C(6)-H(6A)	0.9800
C(6)-H(6B)	0.9800
C(6)-H(6C)	0.9800
C(7)-H(7A)	0.9800
C(7)-H(7B)	0.9800
C(7)-H(7C)	0.9800
C(8)-H(8A)	0.9800
C(8)-H(8B)	0.9800
C(8)-H(8C)	0.9800
C(9)-H(9A)	0.9800
C(9)-H(9B)	0.9800
C(9)-H(9C)	0.9800
C(10)-H(10A)	0.9800
C(10)-H(10B)	0.9800
C(10)-H(10C)	0.9800
C(11)-H(11A)	0.9800
C(11)-H(11B)	0.9800
C(11)-H(11C)	0.9800
C(12)-C(17)	1.391(13)
C(12)-C(13)	1.400(13)
C(13)-C(14)	1.366(12)
C(13)-H(13)	0.9500

C(14)-C(15)	1.410(15)
C(14)-H(14)	0.9500
C(15)-C(16)	1.336(15)
C(15)-H(15)	0.9500
C(16)-C(17)	1.380(13)
C(16)-H(16)	0.9500
C(17)-H(17)	0.9500
C(18)-C(45)	1.526(16)
C(18)-H(18)	1.0000
C(19)-C(20)	1.27(3)
C(19)-C(24)	1.31(3)
C(20)-C(21)	1.32(2)
C(20)-C(28)	1.53(3)
C(21)-C(22)	1.33(3)
C(21)-H(21)	0.9500
C(22)-C(23)	1.52(4)
C(22)-H(22)	0.9500
C(23)-C(24)	1.45(2)
C(23)-H(23)	0.9500
C(24)-C(25)	1.57(3)
C(25)-C(27)	1.48(3)
C(25)-C(26)	1.55(2)
C(25)-H(25)	1.0000
C(26)-H(26A)	0.9800
C(26)-H(26B)	0.9800
C(26)-H(26C)	0.9800
C(27)-H(27A)	0.9800
C(27)-H(27B)	0.9800
C(27)-H(27C)	0.9800
C(28)-C(29)	1.49(2)
C(28)-C(30)	1.53(3)
C(28)-H(28)	1.0000
C(29)-H(29A)	0.9800
C(29)-H(29B)	0.9800
C(29)-H(29C)	0.9800
C(30)-H(30A)	0.9800
C(30)-H(30B)	0.9800
C(30)-H(30C)	0.9800

C(31)-C(32)	1.295(16)
C(31)-H(31)	0.9500
C(32)-H(32)	0.9500
C(33)-C(34)	1.37(2)
C(33)-C(38)	1.37(3)
C(34)-C(35)	1.420(16)
C(34)-C(42)	1.531(19)
C(35)-C(36)	1.374(19)
C(35)-H(35)	0.9500
C(36)-C(37)	1.36(2)
C(36)-H(36)	0.9500
C(37)-C(38)	1.52(2)
C(37)-H(37)	0.9500
C(38)-C(39)	1.35(2)
C(39)-C(41)	1.39(2)
C(39)-C(40)	1.47(2)
C(39)-H(39)	1.0000
C(40)-H(40A)	0.9800
C(40)-H(40B)	0.9800
C(40)-H(40C)	0.9800
C(41)-H(41A)	0.9800
C(41)-H(41B)	0.9800
C(41)-H(41C)	0.9800
C(42)-C(43)	1.55(2)
C(42)-C(44)	1.59(2)
C(42)-H(42)	1.0000
C(43)-H(43A)	0.9800
C(43)-H(43B)	0.9800
C(43)-H(43C)	0.9800
C(44)-H(44A)	0.9800
C(44)-H(44B)	0.9800
C(44)-H(44C)	0.9800
C(45)-C(46)	1.359(18)
C(46)-C(47)	1.23(2)
C(46)-H(46)	0.9500
C(47)-C(48)	1.39(3)
C(47)-H(47)	0.9500
C(48)-C(49)	1.55(3)

C(48)-H(48)	0.9500
C(49)-H(49)	0.9500
C(50)-C(51)	1.51(2)
C(50)-H(50)	0.9500
C(51)-C(52)	1.25(3)
C(51)-H(51)	0.9500
C(52)-C(53)	1.21(3)
C(52)-H(52)	0.9500
C(53)-C(54)	1.382(19)
C(53)-H(53)	0.9500
C(54)-H(54)	0.9500
C(55)-C(60)	1.36(5)
C(55)-C(56)	1.37(2)
C(55)-H(55)	0.9500
C(56)-C(57)	1.35(2)
C(56)-H(56)	0.9500
C(57)-C(58)	1.33(2)
C(57)-H(57)	0.9500
C(58)-C(59)	1.29(4)
C(58)-H(58)	0.9500
C(59)-C(60)	1.27(4)
C(59)-H(59)	0.9500
C(60)-H(60)	0.9500
C(61)-C(62)	1.3900
C(61)-C(66)	1.3900
C(61)-H(61)	0.9500
C(62)-C(63)	1.3900
C(62)-H(62)	0.9500
C(63)-C(64)	1.3900
C(63)-H(63)	0.9500
C(64)-C(65)	1.3900
C(64)-H(64)	0.9500
C(65)-C(66)	1.3900
C(65)-H(65)	0.9500
C(66)-H(66)	0.9500
O(1)-Sc(1)-N(1)	143.2(2)
O(1)-Sc(1)-N(4)	75.5(4)

N(1)-Sc(1)-N(4)	88.5(4)
O(1)-Sc(1)-N(5)	78.9(4)
N(1)-Sc(1)-N(5)	90.0(4)
N(4)-Sc(1)-N(5)	134.4(3)
O(1)-Sc(1)-C(1)	145.4(3)
N(1)-Sc(1)-C(1)	71.2(3)
N(4)-Sc(1)-C(1)	110.5(4)
N(5)-Sc(1)-C(1)	112.0(4)
O(1)-Sc(1)-C(5)	120.9(3)
N(1)-Sc(1)-C(5)	92.4(3)
N(4)-Sc(1)-C(5)	140.9(3)
N(5)-Sc(1)-C(5)	84.6(3)
C(1)-Sc(1)-C(5)	35.3(3)
O(1)-Sc(1)-C(2)	120.8(3)
N(1)-Sc(1)-C(2)	90.3(3)
N(4)-Sc(1)-C(2)	86.3(4)
N(5)-Sc(1)-C(2)	139.3(3)
C(1)-Sc(1)-C(2)	31.9(4)
C(5)-Sc(1)-C(2)	54.7(2)
O(1)-Sc(1)-C(3)	92.5(3)
N(1)-Sc(1)-C(3)	121.9(3)
N(4)-Sc(1)-C(3)	93.7(3)
N(5)-Sc(1)-C(3)	124.7(3)
C(1)-Sc(1)-C(3)	53.9(3)
C(5)-Sc(1)-C(3)	53.5(3)
C(2)-Sc(1)-C(3)	32.4(4)
O(1)-Sc(1)-C(4)	92.6(3)
N(1)-Sc(1)-C(4)	123.3(3)
N(4)-Sc(1)-C(4)	124.7(3)
N(5)-Sc(1)-C(4)	93.3(3)
C(1)-Sc(1)-C(4)	55.2(3)
C(5)-Sc(1)-C(4)	32.1(3)
C(2)-Sc(1)-C(4)	53.8(3)
C(3)-Sc(1)-C(4)	32.0(2)
O(1)-Sc(1)-Si(1)	176.30(18)
N(1)-Sc(1)-Si(1)	33.43(18)
N(4)-Sc(1)-Si(1)	101.8(3)
N(5)-Sc(1)-Si(1)	101.6(3)

C(1)-Sc(1)-Si(1)	37.78(19)
C(5)-Sc(1)-Si(1)	62.8(2)
C(2)-Sc(1)-Si(1)	61.1(3)
C(3)-Sc(1)-Si(1)	90.2(2)
C(4)-Sc(1)-Si(1)	91.0(2)
N(1)-Si(1)-C(11)	114.1(7)
N(1)-Si(1)-C(1)	96.8(3)
C(11)-Si(1)-C(1)	114.2(6)
N(1)-Si(1)-C(10)	114.2(7)
C(11)-Si(1)-C(10)	105.4(8)
C(1)-Si(1)-C(10)	112.3(7)
N(1)-Si(1)-Sc(1)	43.8(2)
C(11)-Si(1)-Sc(1)	126.3(7)
C(1)-Si(1)-Sc(1)	53.1(2)
C(10)-Si(1)-Sc(1)	128.1(6)
N(2)-B(1)-N(3)	104.1(10)
N(2)-B(1)-C(18)	124.3(10)
N(3)-B(1)-C(18)	131.5(10)
C(18)-O(1)-Sc(1)	122.3(6)
C(12)-N(1)-Si(1)	125.3(6)
C(12)-N(1)-Sc(1)	131.7(6)
Si(1)-N(1)-Sc(1)	102.8(3)
B(1)-N(2)-C(31)	108.9(9)
B(1)-N(2)-C(19)	131.2(10)
C(31)-N(2)-C(19)	119.8(9)
C(32)-N(3)-B(1)	108.5(10)
C(32)-N(3)-C(33)	119.1(11)
B(1)-N(3)-C(33)	132.3(11)
C(49)-N(4)-C(45)	123.2(11)
C(49)-N(4)-Sc(1)	127.6(9)
C(45)-N(4)-Sc(1)	108.8(7)
C(50)-N(5)-C(54)	116.2(12)
C(50)-N(5)-Sc(1)	121.6(10)
C(54)-N(5)-Sc(1)	122.1(10)
C(2)-C(1)-C(5)	106.4(7)
C(2)-C(1)-Si(1)	124.1(8)
C(5)-C(1)-Si(1)	120.5(8)
C(2)-C(1)-Sc(1)	75.8(5)

C(5)-C(1)-Sc(1)	73.8(4)
Si(1)-C(1)-Sc(1)	89.1(3)
C(1)-C(2)-C(3)	110.1(9)
C(1)-C(2)-C(9)	128.6(11)
C(3)-C(2)-C(9)	121.1(10)
C(1)-C(2)-Sc(1)	72.4(5)
C(3)-C(2)-Sc(1)	75.7(6)
C(9)-C(2)-Sc(1)	123.7(7)
C(2)-C(3)-C(4)	109.3(10)
C(2)-C(3)-C(8)	124.8(10)
C(4)-C(3)-C(8)	125.3(11)
C(2)-C(3)-Sc(1)	71.9(6)
C(4)-C(3)-Sc(1)	75.8(6)
C(8)-C(3)-Sc(1)	125.4(7)
C(5)-C(4)-C(3)	106.8(10)
C(5)-C(4)-C(7)	127.5(10)
C(3)-C(4)-C(7)	125.7(11)
C(5)-C(4)-Sc(1)	70.1(5)
C(3)-C(4)-Sc(1)	72.2(6)
C(7)-C(4)-Sc(1)	122.4(6)
C(4)-C(5)-C(6)	123.7(9)
C(4)-C(5)-C(1)	107.4(8)
C(6)-C(5)-C(1)	128.6(9)
C(4)-C(5)-Sc(1)	77.8(5)
C(6)-C(5)-Sc(1)	122.5(7)
C(1)-C(5)-Sc(1)	71.0(4)
C(5)-C(6)-H(6A)	109.1
C(5)-C(6)-H(6B)	109.8
H(6A)-C(6)-H(6B)	109.5
C(5)-C(6)-H(6C)	109.5
H(6A)-C(6)-H(6C)	109.5
H(6B)-C(6)-H(6C)	109.5
C(4)-C(7)-H(7A)	109.7
C(4)-C(7)-H(7B)	108.7
H(7A)-C(7)-H(7B)	109.5
C(4)-C(7)-H(7C)	110.1
H(7A)-C(7)-H(7C)	109.5
H(7B)-C(7)-H(7C)	109.5

C(3)-C(8)-H(8A)	110.1
C(3)-C(8)-H(8B)	109.5
H(8A)-C(8)-H(8B)	109.5
C(3)-C(8)-H(8C)	108.9
H(8A)-C(8)-H(8C)	109.5
H(8B)-C(8)-H(8C)	109.5
C(2)-C(9)-H(9A)	108.3
C(2)-C(9)-H(9B)	110.4
H(9A)-C(9)-H(9B)	109.5
C(2)-C(9)-H(9C)	109.6
H(9A)-C(9)-H(9C)	109.5
H(9B)-C(9)-H(9C)	109.5
Si(1)-C(10)-H(10A)	110.1
Si(1)-C(10)-H(10B)	109.2
H(10A)-C(10)-H(10B)	109.5
Si(1)-C(10)-H(10C)	109.1
H(10A)-C(10)-H(10C)	109.5
H(10B)-C(10)-H(10C)	109.5
Si(1)-C(11)-H(11A)	109.9
Si(1)-C(11)-H(11B)	108.9
H(11A)-C(11)-H(11B)	109.5
Si(1)-C(11)-H(11C)	109.6
H(11A)-C(11)-H(11C)	109.5
H(11B)-C(11)-H(11C)	109.5
N(1)-C(12)-C(17)	124.0(8)
N(1)-C(12)-C(13)	121.5(8)
C(17)-C(12)-C(13)	114.4(8)
C(14)-C(13)-C(12)	123.3(9)
C(14)-C(13)-H(13)	118.0
C(12)-C(13)-H(13)	118.7
C(13)-C(14)-C(15)	119.5(10)
C(13)-C(14)-H(14)	120.3
C(15)-C(14)-H(14)	120.2
C(16)-C(15)-C(14)	118.3(8)
C(16)-C(15)-H(15)	120.9
C(14)-C(15)-H(15)	120.7
C(15)-C(16)-C(17)	121.4(9)
C(15)-C(16)-H(16)	119.3

C(17)-C(16)-H(16)	119.3
C(16)-C(17)-C(12)	122.5(9)
C(16)-C(17)-H(17)	118.7
C(12)-C(17)-H(17)	118.9
O(1)-C(18)-C(45)	109.3(8)
O(1)-C(18)-B(1)	113.0(8)
C(45)-C(18)-B(1)	117.9(9)
O(1)-C(18)-H(18)	105.0
C(45)-C(18)-H(18)	104.8
B(1)-C(18)-H(18)	105.4
C(20)-C(19)-C(24)	128.0(16)
C(20)-C(19)-N(2)	118.3(19)
C(24)-C(19)-N(2)	113(2)
C(19)-C(20)-C(21)	113.8(19)
C(19)-C(20)-C(28)	126.9(15)
C(21)-C(20)-C(28)	118.9(19)
C(20)-C(21)-C(22)	130.0(19)
C(20)-C(21)-H(21)	116.6
C(22)-C(21)-H(21)	113.5
C(21)-C(22)-C(23)	114.6(13)
C(21)-C(22)-H(22)	123.4
C(23)-C(22)-H(22)	122.0
C(24)-C(23)-C(22)	112.0(17)
C(24)-C(23)-H(23)	125.7
C(22)-C(23)-H(23)	122.3
C(19)-C(24)-C(23)	121(2)
C(19)-C(24)-C(25)	129.9(17)
C(23)-C(24)-C(25)	109(2)
C(27)-C(25)-C(26)	110.2(17)
C(27)-C(25)-C(24)	110.4(14)
C(26)-C(25)-C(24)	114.9(17)
C(27)-C(25)-H(25)	107.2
C(26)-C(25)-H(25)	108.4
C(24)-C(25)-H(25)	105.3
C(25)-C(26)-H(26A)	107.0
C(25)-C(26)-H(26B)	111.3
H(26A)-C(26)-H(26B)	109.5
C(25)-C(26)-H(26C)	110.1

H(26A)-C(26)-H(26C)	109.5
H(26B)-C(26)-H(26C)	109.5
C(25)-C(27)-H(27A)	108.6
C(25)-C(27)-H(27B)	110.3
H(27A)-C(27)-H(27B)	109.5
C(25)-C(27)-H(27C)	109.6
H(27A)-C(27)-H(27C)	109.5
H(27B)-C(27)-H(27C)	109.5
C(29)-C(28)-C(30)	108.7(18)
C(29)-C(28)-C(20)	113.1(15)
C(30)-C(28)-C(20)	113.7(14)
C(29)-C(28)-H(28)	108.0
C(30)-C(28)-H(28)	107.8
C(20)-C(28)-H(28)	105.2
C(28)-C(29)-H(29A)	108.3
C(28)-C(29)-H(29B)	110.7
H(29A)-C(29)-H(29B)	109.5
C(28)-C(29)-H(29C)	109.4
H(29A)-C(29)-H(29C)	109.5
H(29B)-C(29)-H(29C)	109.5
C(28)-C(30)-H(30A)	110.7
C(28)-C(30)-H(30B)	109.1
H(30A)-C(30)-H(30B)	109.5
C(28)-C(30)-H(30C)	108.6
H(30A)-C(30)-H(30C)	109.5
H(30B)-C(30)-H(30C)	109.5
C(32)-C(31)-N(2)	108.0(10)
C(32)-C(31)-H(31)	126.0
N(2)-C(31)-H(31)	126.0
C(31)-C(32)-N(3)	110.4(10)
C(31)-C(32)-H(32)	124.9
N(3)-C(32)-H(32)	124.6
C(34)-C(33)-C(38)	125.3(14)
C(34)-C(33)-N(3)	121.1(15)
C(38)-C(33)-N(3)	113.4(16)
C(33)-C(34)-C(35)	119.2(12)
C(33)-C(34)-C(42)	123.2(12)
C(35)-C(34)-C(42)	117.3(11)

C(36)-C(35)-C(34)	119.8(12)
C(36)-C(35)-H(35)	120.3
C(34)-C(35)-H(35)	119.9
C(37)-C(36)-C(35)	121.0(11)
C(37)-C(36)-H(36)	119.5
C(35)-C(36)-H(36)	119.4
C(36)-C(37)-C(38)	121.0(15)
C(36)-C(37)-H(37)	120.0
C(38)-C(37)-H(37)	118.9
C(39)-C(38)-C(33)	133.9(18)
C(39)-C(38)-C(37)	109(2)
C(33)-C(38)-C(37)	113.3(16)
C(38)-C(39)-C(41)	123.6(16)
C(38)-C(39)-C(40)	128.9(17)
C(41)-C(39)-C(40)	104.1(12)
C(38)-C(39)-H(39)	96.8
C(41)-C(39)-H(39)	95.0
C(40)-C(39)-H(39)	96.3
C(39)-C(40)-H(40A)	108.2
C(39)-C(40)-H(40B)	109.8
H(40A)-C(40)-H(40B)	109.5
C(39)-C(40)-H(40C)	110.3
H(40A)-C(40)-H(40C)	109.5
H(40B)-C(40)-H(40C)	109.5
C(39)-C(41)-H(41A)	108.5
C(39)-C(41)-H(41B)	110.3
H(41A)-C(41)-H(41B)	109.5
C(39)-C(41)-H(41C)	109.6
H(41A)-C(41)-H(41C)	109.5
H(41B)-C(41)-H(41C)	109.5
C(34)-C(42)-C(43)	113.7(14)
C(34)-C(42)-C(44)	111.1(14)
C(43)-C(42)-C(44)	107.2(15)
C(34)-C(42)-H(42)	108.8
C(43)-C(42)-H(42)	109.1
C(44)-C(42)-H(42)	106.7
C(42)-C(43)-H(43A)	108.5
C(42)-C(43)-H(43B)	109.7

H(43A)-C(43)-H(43B)	109.5
C(42)-C(43)-H(43C)	110.2
H(43A)-C(43)-H(43C)	109.5
H(43B)-C(43)-H(43C)	109.5
C(42)-C(44)-H(44A)	111.6
C(42)-C(44)-H(44B)	107.6
H(44A)-C(44)-H(44B)	109.5
C(42)-C(44)-H(44C)	109.2
H(44A)-C(44)-H(44C)	109.5
H(44B)-C(44)-H(44C)	109.5
C(46)-C(45)-N(4)	117.6(12)
C(46)-C(45)-C(18)	125.6(11)
N(4)-C(45)-C(18)	116.7(10)
C(47)-C(46)-C(45)	121.2(14)
C(47)-C(46)-H(46)	120.3
C(45)-C(46)-H(46)	118.5
C(46)-C(47)-C(48)	129.2(16)
C(46)-C(47)-H(47)	114.7
C(48)-C(47)-H(47)	116.1
C(47)-C(48)-C(49)	111.3(15)
C(47)-C(48)-H(48)	123.2
C(49)-C(48)-H(48)	125.4
N(4)-C(49)-C(48)	117.2(16)
N(4)-C(49)-H(49)	121.8
C(48)-C(49)-H(49)	121.0
N(5)-C(50)-C(51)	116.3(15)
N(5)-C(50)-H(50)	121.8
C(51)-C(50)-H(50)	121.9
C(52)-C(51)-C(50)	114.6(18)
C(52)-C(51)-H(51)	120.8
C(50)-C(51)-H(51)	124.6
C(53)-C(52)-C(51)	135(2)
C(53)-C(52)-H(52)	110.9
C(51)-C(52)-H(52)	114.1
C(52)-C(53)-C(54)	107.2(19)
C(52)-C(53)-H(53)	127.0
C(54)-C(53)-H(53)	125.9
N(5)-C(54)-C(53)	129.5(18)

N(5)-C(54)-H(54)	115.5
C(53)-C(54)-H(54)	115.0
C(60)-C(55)-C(56)	121(3)
C(60)-C(55)-H(55)	115.9
C(56)-C(55)-H(55)	123.3
C(57)-C(56)-C(55)	117(3)
C(57)-C(56)-H(56)	125.4
C(55)-C(56)-H(56)	118.0
C(58)-C(57)-C(56)	119(2)
C(58)-C(57)-H(57)	123.0
C(56)-C(57)-H(57)	118.2
C(59)-C(58)-C(57)	120(3)
C(59)-C(58)-H(58)	122.9
C(57)-C(58)-H(58)	116.8
C(60)-C(59)-C(58)	125(3)
C(60)-C(59)-H(59)	120.9
C(58)-C(59)-H(59)	114.6
C(59)-C(60)-C(55)	117(3)
C(59)-C(60)-H(60)	119.1
C(55)-C(60)-H(60)	123.8
C(62)-C(61)-C(66)	120.0
C(62)-C(61)-H(61)	126.8
C(66)-C(61)-H(61)	113.1
C(63)-C(62)-C(61)	120.0
C(63)-C(62)-H(62)	120.0
C(61)-C(62)-H(62)	120.0
C(62)-C(63)-C(64)	120.0
C(62)-C(63)-H(63)	120.0
C(64)-C(63)-H(63)	120.0
C(63)-C(64)-C(65)	120.0
C(63)-C(64)-H(64)	120.0
C(65)-C(64)-H(64)	120.0
C(66)-C(65)-C(64)	120.0
C(66)-C(65)-H(65)	120.0
C(64)-C(65)-H(65)	120.0
C(65)-C(66)-C(61)	120.0
C(65)-C(66)-H(66)	120.0
C(61)-C(66)-H(66)	120.0

Symmetry transformations used to generate equivalent atoms:

Table S4. Anisotropic displacement parameters ($Å^2$x 10^3)for complex **5**. The anisotropic displacement factor exponent takes the form: $-2\pi^2[h^2a^{*2}U^{11} + ... + 2 h k a^* b^* U^{12}]$

	U^{11}	U^{22}	U^{33}	U^{23}	U^{13}	U^{12}
Sc(1)	19(1)	20(1)	37(1)	-2(1)	5(1)	0(1)
Si(1)	32(1)	88(2)	39(1)	10(2)	6(1)	7(2)
B(1)	33(6)	45(7)	69(8)	-4(6)	5(6)	-2(5)
O(1)	37(3)	17(2)	40(3)	-16(4)	7(2)	-9(3)
N(1)	23(3)	42(5)	50(4)	2(5)	3(3)	-7(4)
N(2)	45(5)	66(6)	50(5)	-13(5)	6(4)	-9(4)
N(3)	57(6)	76(7)	53(6)	-3(5)	12(5)	-7(5)
N(4)	32(5)	42(6)	70(6)	16(5)	12(5)	-1(4)
N(5)	25(4)	23(5)	99(7)	-8(5)	38(5)	0(4)
C(1)	26(4)	40(5)	39(4)	-4(6)	16(3)	-12(6)
C(2)	29(5)	32(6)	56(7)	-3(6)	15(5)	0(5)
C(3)	19(5)	26(6)	51(6)	4(5)	14(5)	3(4)
C(4)	23(5)	33(7)	44(6)	3(5)	13(4)	-3(4)
C(5)	22(5)	19(5)	42(5)	7(4)	15(4)	-2(4)
C(6)	39(6)	42(7)	61(7)	9(6)	16(5)	-3(5)
C(7)	35(6)	59(8)	55(7)	-16(6)	17(5)	-18(6)
C(8)	41(6)	36(6)	82(8)	10(6)	13(6)	11(5)
C(9)	56(8)	28(7)	87(9)	-25(6)	26(7)	-10(5)
C(10)	75(10)	144(16)	48(7)	-41(9)	3(7)	1(10)
C(11)	34(7)	164(18)	84(10)	67(11)	-10(7)	10(9)
C(12)	31(4)	38(5)	53(5)	-1(6)	2(4)	10(6)
C(13)	29(4)	56(6)	66(6)	1(8)	4(4)	16(6)
C(14)	33(5)	59(6)	92(7)	-2(9)	20(5)	4(7)
C(15)	22(4)	41(5)	78(6)	22(7)	-2(4)	-12(6)
C(16)	32(5)	68(7)	76(7)	-4(9)	-15(5)	16(7)
C(17)	29(4)	91(9)	50(5)	7(7)	0(4)	15(7)
C(18)	43(5)	31(5)	53(6)	-3(5)	4(4)	-6(4)
C(19)	53(8)	180(20)	40(7)	-4(9)	10(6)	-59(11)
C(20)	49(8)	134(17)	69(9)	-29(10)	8(7)	-18(9)
C(21)	61(8)	122(14)	77(9)	-40(9)	41(7)	-19(9)

C(22)	41(6)	157(16)	84(9)	33(13)	7(6)	-39(11)
C(23)	300(40)	150(20)	77(12)	19(14)	-33(18)	-160(30)
C(24)	98(13)	85(13)	54(8)	-7(8)	-23(8)	19(11)
C(25)	108(13)	97(12)	68(9)	17(8)	4(8)	-34(10)
C(26)	180(20)	75(12)	83(11)	10(9)	5(12)	-3(12)
C(27)	121(17)	116(16)	136(17)	10(13)	33(14)	9(13)
C(28)	69(8)	83(10)	63(8)	-20(7)	12(6)	0(7)
C(29)	160(19)	68(11)	136(16)	-40(11)	32(14)	13(12)
C(30)	270(30)	133(19)	62(10)	8(10)	-3(14)	-70(20)
C(31)	53(7)	114(11)	45(6)	-18(7)	3(5)	-18(7)
C(32)	43(6)	118(12)	53(7)	-21(7)	24(5)	-29(7)
C(33)	38(7)	92(12)	93(10)	-29(9)	10(7)	1(7)
C(34)	45(6)	51(7)	72(7)	-5(6)	8(5)	-7(5)
C(35)	38(6)	72(8)	68(7)	4(6)	-4(5)	2(6)
C(36)	44(6)	83(10)	67(7)	1(7)	-2(6)	-15(7)
C(37)	41(6)	83(10)	81(9)	21(7)	-3(6)	-9(6)
C(38)	118(15)	150(20)	44(8)	8(9)	15(8)	-55(13)
C(39)	107(12)	44(9)	74(9)	-18(7)	-18(9)	48(9)
C(40)	78(9)	74(10)	96(10)	-9(8)	28(8)	6(8)
C(41)	52(8)	39(8)	200(20)	15(10)	4(10)	-13(6)
C(42)	55(8)	71(9)	108(11)	9(8)	10(7)	-3(7)
C(43)	61(9)	71(11)	210(20)	-16(12)	36(11)	-21(8)
C(44)	145(19)	138(18)	83(11)	21(12)	3(11)	-20(14)
C(45)	32(6)	63(8)	64(7)	22(6)	22(5)	2(5)
C(46)	94(10)	48(8)	65(8)	4(7)	25(7)	22(7)
C(47)	114(13)	49(8)	66(9)	7(7)	28(8)	-15(8)
C(48)	101(14)	101(15)	139(17)	-72(13)	40(13)	4(11)
C(49)	78(9)	24(6)	117(11)	-26(7)	60(8)	-29(6)
C(50)	40(6)	66(9)	108(11)	-71(9)	17(7)	-17(6)
C(51)	71(10)	87(12)	110(13)	-29(11)	35(9)	-17(9)
C(52)	97(14)	79(13)	170(20)	55(13)	-51(14)	-51(11)
C(53)	67(9)	39(8)	152(16)	14(9)	47(10)	14(7)
C(54)	80(10)	34(7)	120(12)	34(7)	62(9)	25(7)
C(55)	150(30)	170(30)	200(30)	-100(30)	40(20)	-20(20)
C(56)	89(15)	190(30)	66(12)	3(14)	-14(10)	-38(17)
C(57)	49(9)	180(20)	105(16)	-48(17)	27(9)	6(13)
C(58)	98(15)	150(20)	89(14)	-3(14)	-17(12)	34(14)
C(59)	110(20)	210(30)	110(20)	60(20)	3(17)	-20(20)

C(60)	92(15)	340(60)	72(12)	-70(20)	23(10)	-70(30)

Computational Section

The complexes **2** (Figure S7) and **2-^{13}C** were optimized by the DFT method of M06[6], which was implanted in Gaussian 09 program.[7] In these calculations, the 6-31G* basis set was used for C, H, O, N, and B atoms, and the Sc and Si atoms were treated by the Stuttgart/Dresden effective core potential (ECP) and the associated basis sets.[8] In the Stuttgart/Dresden ECP used in this study, the most inner 10 electrons of Si and Sc are included in the core. The 4 valence electrons of Si atom and 11 valence electrons of Sc were treated by the optimized basis sets, viz. (4s4p)/[2s2p] for Si and (8s7p6d1f)/[6s5p3d1f] for Sc, respectively. The basis set for Sc atom contains one f-polarization function with exponent of 0.27. One d-polarization function (exponent of 0.28) was augmented for Si atom. The optimized structures were analyzed by harmonic vibrational frequencies obtained at the same level and characterized as a minimum (N_{imag} = 0). The recommended scale factors for frequencies in M06/6-31+G** level is 0.950.[9] The second order perturbation theory analysis was performed with the basis set 6-31+G** for C, H, O, N, and B atoms and the basis sets for Sc and Si are same as those in geometry optimizations. The calculated structure showed excellent agreement with the crystallographic structure (Table S5).

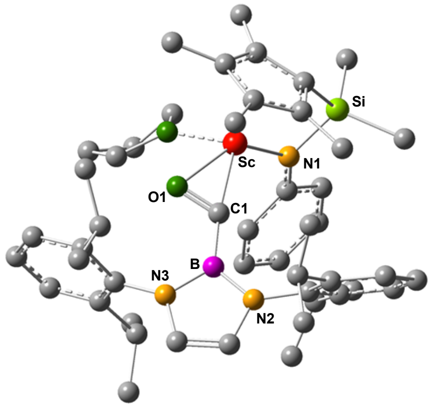

Figure S7. Optimized structure of complex **2**.

Table S5. Structural comparison between X-ray structure and DFT optimized structure of complex **2** (bond distance in Å and angle in degree)

Parameters	DFT optimized structure	X-ray structure
Sc–C1	2.173	2.194
Sc–O1	2.119	2.115
C1–O1	1.271	1.266
C1–B	1.558	1.577
Sc–N1	2.109	2.130
Sc–C1–O1	70.48	69.49
Sc–O1–C1	75.11	76.40
Sc–C1–B	172.04	172.10
Sc–N1–Si	101.51	102.25

It was found that the calculated stretching frequency is close to that of experiment data (Table S6). The bond stretch of the C1–O1 unit in complex **2** could not be identified in the

experimentally recorded IR spectrum because of an overlap with those of the Cp and boryl moieties. The DFT analyses were therefore performed, showing the C1–O1 stretching frequency of complex **2** at 1450cm^{-1} and of complex **2-^{13}C** at 1417 cm^{-1}.

{IR value= (value in Figures S8, S9)×0.95}

Table S6. Selected calculated and experimental IR data for **2** and **2-^{13}C**.

complex	vibration	computation	experiment	Δ = |comp.–exp.|
**2**	C(Ph)–N1(Si)	1279	1285	6
	Boryl ring	1367	1391	24
	C(Ph)–N2(Boryl)	1439	1444	5
	C=C (on Ph–N1)	1453	1488	19
	C=C (on Ph–N3/1)	1575	1587	4
	C1–O1 (acyl)	1450	–	–
**2-^{13}C**	C1–O1 (acyl)	1417	–	–

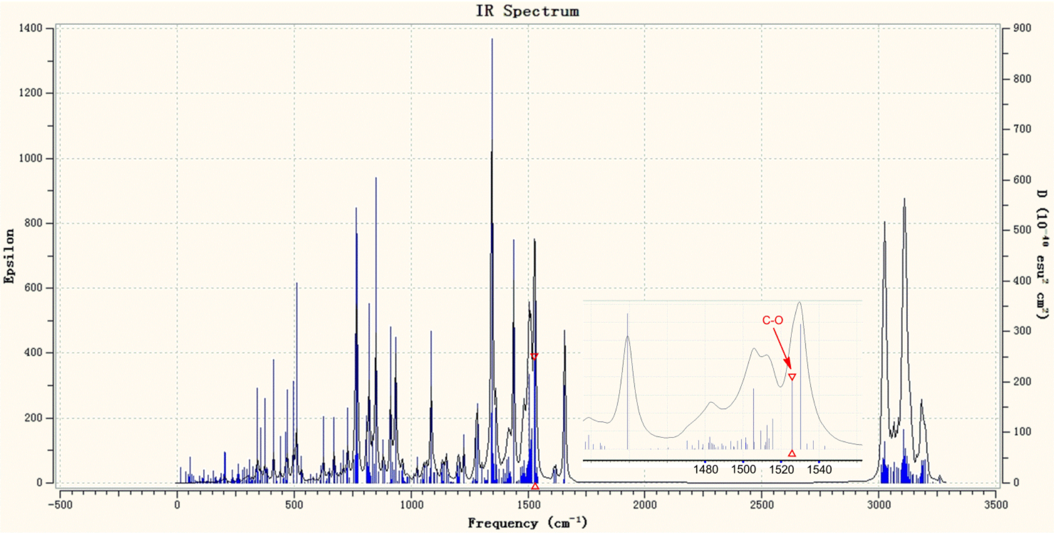

Figure S8. Computational IR spectrum of complex **2**.

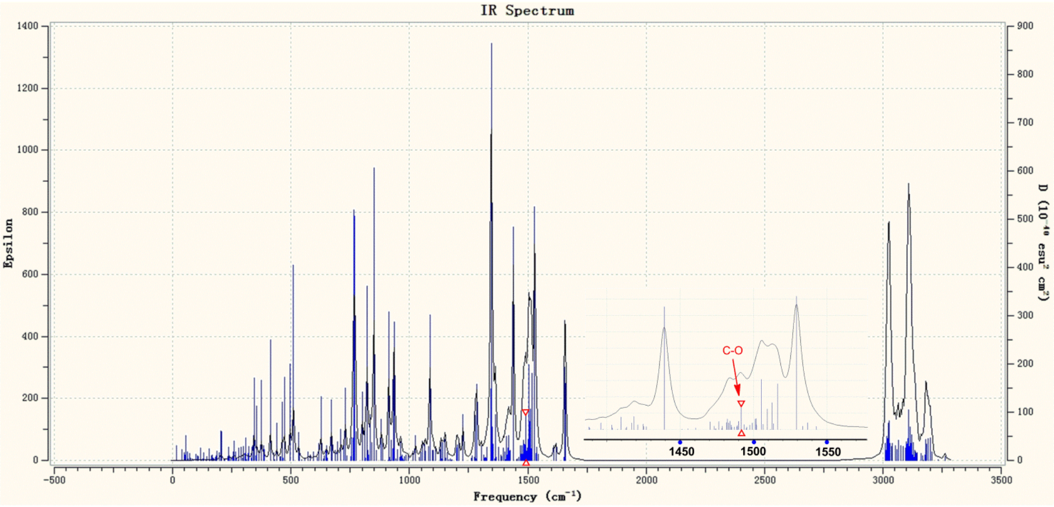

Figure S9. Computational IR spectrum of **2-^{13}C**.

Table S7. Second order perturbation theory analysis for **2**

Donor	Acceptor		E/kcal mol^{-1}
σ(O1–C1)	Sc	LP*3	21.46
		LP*4	30.39
		LP*5	12.76
		LP*8	19.43
		LP*9	11.93
		Total donation	**96.0**
σ(B–C1)	Sc	LP*3	18.34
		LP*4	23.41
		LP*5	23.15
		LP*6	10.13
		LP*8	83.30
		LP*9	19.36
		Total donation	**177.7**
LP(1) O1	Sc	LP*8	24.71
		Total donation	**24.7**
LP(2) O1	Sc	LP*3	14.44
		LP*4	33.88
		LP*5	10.28
		Total donation	**58.6**
LP(1) C1	Sc	LP*1	141.28
		LP*3	14.87
		LP*4	14.14
		Total donation	**170.3**
LP(1) N1	LP*(B)		**57.9**
LP(1) N2	LP*(B)		**88.8**

References:

(1) B. Wang, M. Nishiura, J. Cheng and Z. Hou, *Dalton Trans.*, 2014, 14215.

(2) SMART Software, Version 4.21; Bruker AXS, Inc.: Madison, WI, 1997.

(3) SAINT, Version 6.45; Bruker AXS, Inc.: Madison, WI 2003.

(4) G. M. Sheldrick, SADABS, Version 2.10; Bruker AXS, Inc.: Madison, WI 2003.

(5) G. M. Sheldrick, Acta Xrystallogr., 2008, **A64**, 112.

(6) Y. Zhao and D. G. Truhlar, *Theor Chem Account*, 2008, **120**, 215.

(7) M. Frisch *et al*, *Gaussian 09, Revision A.02*; Gaussian Inc.: Wallingford CT, 2009.

(8) (a) M. Dolg, U. Wedig, H. Stoll and H. Preuss, *J. Chem. Phys.*, 1987, **86**, 866; (b) P. Schwerdtfeger, M. Dolg, W. Schwarz, G. Bowmaker, P. Boyd, *J. Chem. Phys.*, 1989, **91**, 1762; (c) M. Dolg, H. Stoll, A. Savin and H. Preuss, *Theor. Chim. Acta.*, 1989, **75**, 173; (d) D. Andrae, U. Haeussermann, M. Dolg, H. Stoll and H. Preuss, *Theor. Chim. Acta.*, 1990, **77**, 123; (e) M. Dolg, H. Stoll and H. Preuss, *Theor. Chim. Acta.*, 1993, **85**, 441; (f) A. Bergner, M. Dolg, W. Kuechle, H. Stoll and H. Preuss, *Mol. Phys.*, 1993, **80**, 1431.

(9) I. M. Alecu, J. Zheng, Y. Zhao and D.G. Truhlar, *J. Chem. Theory Comput.*, 2010, **6**, 2872.
